# Integrative transcriptomic analysis reveals cross-species conserved core genes and pathways in alveolar macrophages during ALI/ARDS

**DOI:** 10.1186/s12890-025-03928-y

**Published:** 2025-10-02

**Authors:** Aguo Li, Kenqi Zhang, Hongyan Wang, Jianhua Li, Yeping Yao, Yong-Sheng Tu

**Affiliations:** 1 The Second Clinical College, Guangzhou Medical University, Guangzhou, 511436 China; 2 School of Public Health, Guangzhou Medical University, Guangzhou, 511436 China; 3 Department of Pathology, School of Basic Medical Sciences, Guangzhou Medical University, Guangzhou, 511436 China; 4 State Key Laboratory of Respiratory disease, Key Laboratory of Protein Modification and Degradation, School of Basic Medical Sciences, Guangzhou Medical University, Guangzhou, 511436 Guangdong China; 5 Department of Infectious Disease, The Fourth Affiliated Hospital of Guangzhou Medical University, Guangzhou, 511300 China; 6 State Key Laboratory of Respiratory disease, Key Laboratory of Protein Modification and Degradation, Department of Physiology, School of Basic Medical Sciences, Guangzhou Medical University, Guangzhou, 511436 China

**Keywords:** Acute lung injury, Alveolar macrophages, Transcriptome sequencing, Bioinformatics analysis, Efferocytosis

## Abstract

**Background and purpose:**

acute respiratory distress syndrome (ARDS) is a severe pulmonary condition characterized by alveolar-capillary damage and refractory hypoxemia. Alveolar macrophages (AMs) play a crucial role in regulating inflammation and repair processes during acute lung injury (ALI)/ARDS. However, the transcriptional and functional changes in AMs during ALI/ARDS remain poorly understood, especially when considering species-specific differences between murine models and human pathophysiology. This study aims to elucidate these changes in AMs during ALI/ARDS by integrating in vitro and cross-species transcriptomic analyses.

**Methods:**

We conducted RNA sequencing on LPS-stimulated MH-S cells and integrated the data with publicly available murine (GSE225406) and human (GSE40885) AM datasets to identify conserved differentially expressed genes (DEGs). Functional enrichment analysis and protein-protein interaction (PPI) network analysis were performed to explore the underlying mechanisms. Core genes were identified and validated using qRT-PCR, Western blot, immunohistochemical staining, and immunofluorescence staining. Additionally, we analyzed the diagnostic potential of these core genes using clinical datasets (GSE121871 and GSE243066).

**Results:**

We identified 45 conserved upregulated genes and 4 downregulated genes across species, highlighting core transcriptional regulators of LPS-induced Macrophage activation. Functional enrichment analysis revealed significant involvement of immune-inflammatory pathways. PPI network analysis identified 10 core genes potentially central to AM-mediated ALI/ARDS pathogenesis. Experimental validation confirmed the upregulation of key genes and demonstrated that LPS treatment significantly impaired the efferocytosis capacity of AMs with dysregulated expression of stabilin-2, suggesting a potential association with this functional defect. Furthermore, the core gene set showed diagnostic potential in ARDS patient samples (AUC = 0.86).

**Conclusion:**

This analysis identifies cross-species conserved core genes and inflammatory pathways in AMs during ALI/ARDS. Our findings provide insights into AM-mediated inflammatory mechanisms and highlight candidate genes for further functional studies to explore their potential as therapeutic targets.

**Supplementary Information:**

The online version contains supplementary material available at 10.1186/s12890-025-03928-y.

## Introduction

ALI is characterized by damage to the alveolar-capillary endothelium and epithelial cells, triggering inflammatory responses in alveolar epithelial cells and alveolar septa, which subsequently lead to alveolar edema and pulmonary interstitial infiltration [1]. ARDS Manifests as refractory hypoxemia and dyspnea, with a mortality rate as high as 25%−45% [2]. A global multicenter prospective study involving 459 intensive care units (ICUs) revealed that ARDS accounted for 10.4% of ICU admissions, with the unadjusted ICU and hospital mortality being 35.3% and 40.0%, respectively, and both augmented with increased ARDS severity [3]. Survivors often face long-term pulmonary dysfunction, including chronic respiratory failure and pulmonary fibrosis [4]. Despite advances in treatment strategies such as mechanical ventilation, fluid management, and supportive care, there is currently no specific drug that significantly reduces ARDS mortality [5]. Given the high morbidity and mortality associated with ALI/ARDS, identifying potential molecular mechanisms underlying these conditions is of critical importance.

AMs are the primary resident phagocytes in lungs, constituting approximately 90% of alveolar immune cells under steady-state conditions. They serve as the first line of defense against respiratory pathogens and play a pivotal role in regulating immune-inflammatory responses [6, 7]. AMs not only phagocytose inhaled pathogens, particulate matter, and cellular debris but also secrete a wide array of molecules, including growth factors, prostaglandins, interleukins (IL), complements, and tumor necrosis factor (TNF), which are crucial in the pathogenesis of ALI [1]. Despite their central role in lung inflammation, the specific transcriptional and functional changes in AMs during ALI/ARDS remain poorly understood, particularly in the context of species-specific differences between murine models and human pathophysiology. Mouse alveolar macrophage cell line cells (MH-S) are mouse AMs transformed by SV-40, retaining some characteristics of AMs, such as the response to Gram-positive bacteria, NOD2 ligand Pam3CSK4, and TLR2 ligand, etc. However, its immune response may not be as comprehensive as that of wild-type alveolar macrophages (AMs) [8]. For instance, wild-type AMs may exhibit a stronger pro-inflammatory response (such as the secretion of IL-1β and TNF-α) after infection, whereas the response of MH-S cells may be more inclined toward specific antiviral or antibacterial mechanisms [8]. MH-S cells can be cultured for a long time under standard culture conditions, and are easy to operate and maintain, without relying on a specific animal model. Therefore, we used LPS-induced MH-S cells to construct an in vitro model for transcriptome sequencing.

Previous studies have attempted to characterize AMs’ transcriptional signatures in ALI/ARDS using human datasets [9, 10] and murine models [11]. For example, researchers compared AMs from ARDS patients with peripheral blood mononuclear cells (from GSE89953) and neutrophils and whole blood cells from healthy controls (GSE76293 and GSE32707). Another study used bronchoscopic saline or LPS challenge in healthy volunteers, collecting AMs 6 h post-challenge for whole-genome transcriptional analysis. This study identified 2,932 differentially expressed genes [9]. While this approach provided valuable insights, it was limited by the use of human volunteers and the complexity of in vivo models. Additionally, the majority of research has been conducted in animal models; however, the direct applicability of these findings to human ARDS is limited by interspecies differences. For instance, RNA sequencing of isolated AMs from bronchoalveolar lavage fluid (BALF) of C57BL/6 mice following intratracheal injection of LPS revealed that resident AMs predominantly exhibited an anti-inflammatory and tissue-reparative transcriptional signature, whereas recruited macrophages displayed a robust pro-inflammatory phenotype [11]. However, these findings may not fully capture the nuances of human AM behaviour.

These investigations were limited by mismatched control cell types (e.g., neutrophils and whole blood cells) in human studies [10] and interspecies variations [9], raising concerns about translational relevance. To address these limitations, we employed transcriptome sequencing to analyse the gene expression profiles and pathway activation of AMs in an LPS-induced in vitro model using MH-S cells [8]. Furthermore, by integrating transcriptomic data from our LPS-treated MH-S cells with publicly available murine (GSE225406) [11] and human (GSE40885) [12] AMs datasets, we aimed to identify conserved inflammatory pathways across species and species-specific responses. Our study not only provides a comprehensive understanding of AMs-mediated molecular mechanisms in ALI/ARDS but also offers potential targets for diagnosis, treatment, and prognosis.

Further, to simulate the heterogeneity of primary AMs and the human microenvironment as closely as possible, we selected the high-virulence Pseudomonas aeruginosa-infected group and the untreated group from GSE121871 [13] for partial validation. Pseudomonas aeruginosa, a common pathogen in hospital-acquired pneumonia and ventilator-associated pneumonia (VAP) [14], causes alveolar barrier disruption and hyperinflammatory responses. Additionally, despite the lack of publicly available AMs datasets comparing healthy individuals and ARDS patients, we used GSE243066 [15] (ARDS patient peripheral blood mononuclear cell sequencing data) for alternative validation, given its clinical phenotype relevance. Although peripheral blood monocytes (PBMs) and AMs differ in functions (local vs. systemic immune regulation) [16], previous studies have shown that in ARDS, PBM recruitment leads to an expansion of the resident AM pool, with over 85% of resident AMs replaced by bone marrow-derived PBMs within two months in mice after LPS treatment [17]. Thus, PBMs’ systemic inflammatory features indirectly reflect ARDS pathological progression.

Finally, to preliminarily assess the clinical diagnostic potential of the core genes, we performed gene set variation analysis (GSVA) based on these genes, calculating enrichment scores across groups within the core gene set. Using these scores as diagnostic markers, we constructed a multi-gene scoring model.

## Materials and methods

### Research approach

**Fig. 1:**
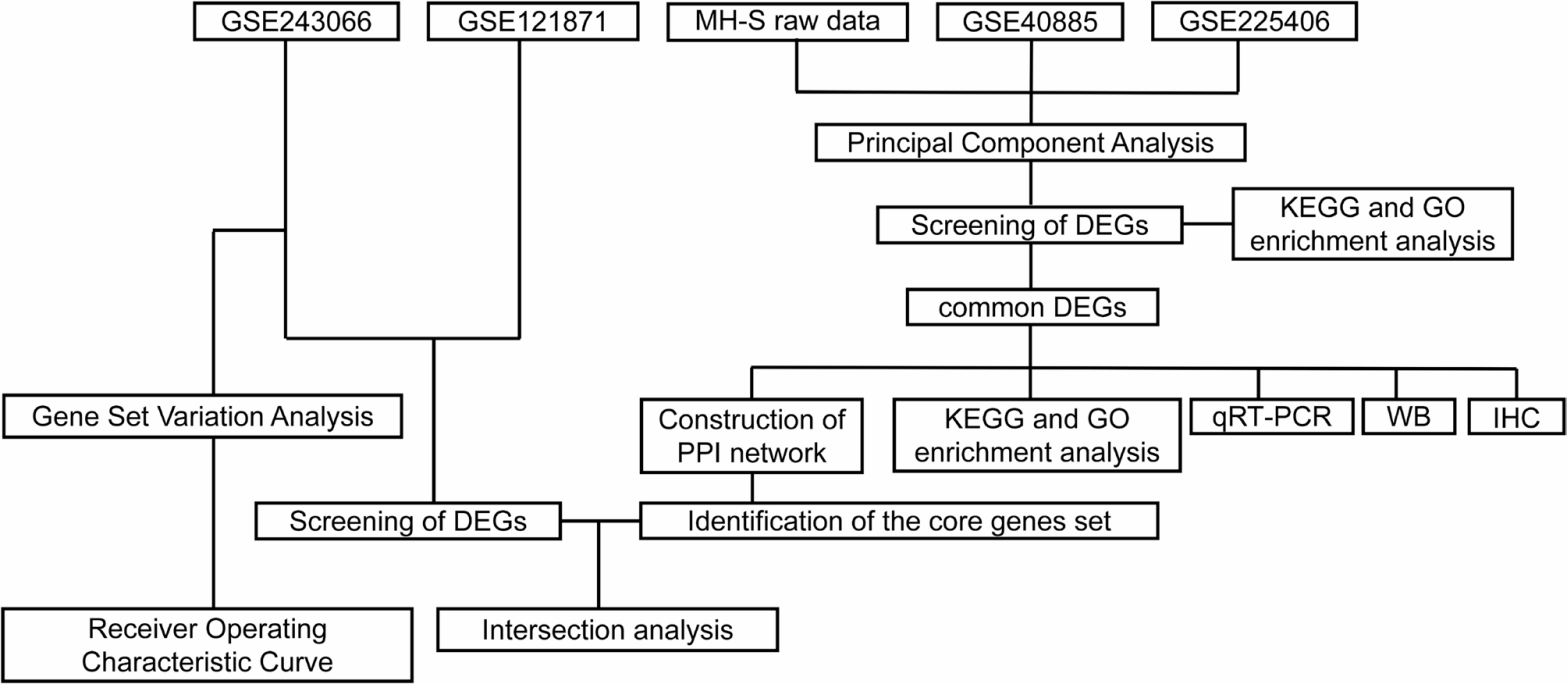
Workflow chart of the study

This study aims to analyse the transcriptional changes of AMs in ALI/ARDS. Firstly, an in vitro model of LPS-stimulated MH-S cells was established. RNA sequencing was performed to identify DEGs, which were then cross-species analysed in combination with mouse (GSE225406) and human (GSE40885) AMs datasets to screen for conserved DEGs. Subsequently, functional enrichment analysis was carried out to reveal immune-inflammatory pathways, and a PPI network was constructed to identify core genes. The expression of key genes was validated using qRT-PCR, Western blotting, and other experimental methods. Meanwhile, an in vitro LPS-stimulated MH-S cell model was utilized to assess the altered efferocytosis function of AMs. Finally, datasets (GSE121871 and GSE243066) were integrated, and Gene Set Variation Analysis (GSVA) was performed to evaluate the diagnostic effectiveness of the core genes, establishing a research framework that bridges basic research and clinical translation. The workflow of the study is shown in Fig. 1.

### Transcriptome sample extraction and detection

First, MH-S cells (obtained from ATCC) were seeded in six 10 cm² culture dishes (3 dishes per group), divided into two experimental groups: infection group and control group. When cell density reached 80%−90% on the second day, the experimental group was treated with 10 ng/mL LPS (Millipore Sigma, cat#: L2630) while the control group received an equivalent volume of complete medium. After 72 h of incubation at 37 °C with 5% CO₂, the medium was discarded and cells were washed twice with PBS. Total RNA was extracted using the Cell/Bacterial Total RNA Extraction Kit (Genesand, cat#: RE716). Control samples were designated as MH.S.Normal.1, MH.S.Normal.2, MH.S.Normal.3, while experimental samples were labeled MH.S.LPS.72 h.1, MH.S.LPS.72 h.2, MH.S.LPS.72 h.3. RNA purity, concentration, and integrity were assessed using the Agilent 4200 Bioanalyzer, with samples achieving an RNA Integrity Number (RIN) ≥ 4 qualifying for subsequent analysis.

### Library construction and transcriptome sequencing

After the RNA samples passed quality inspection, eukaryotic mRNA was enriched using magnetic beads with Oligo(dT). Subsequently, the mRNA was fragmented into short segments by adding a fragmentation buffer. Using the mRNA as a template, single-stranded cDNA was synthesized with random hexamer primers. Then, buffer, dNTPs, DNA polymerase I, and RNase H were added to synthesize double-stranded cDNA, which was subsequently purified using AMPure XP beads. The purified double-stranded cDNA underwent end repair, A-tailing, and sequencing adapter ligation, followed by size selection using AMPure XP beads. Finally, PCR amplification was performed, and the PCR products were purified with AMPure XP beads to obtain the final library. The constructed library underwent quality control, and qualified libraries were subjected to PE150 sequencing using the Illumina HiSeq 2500 high-throughput sequencing platform.

### Sequencing data quality control

After obtaining the sequencing data of the sample, the first step was to assess the quality of the sequencing data and filter out low-quality data to ensure the reliability of the subsequent analysis results. The quality control (QC) process uses the software fastp. The QC criteria were as follows: 1. The proportion of bases with quality values lower than 15 should not exceed 40%; 2. Starting from the 5’ end of the read, in a sliding window of 4 bases, the average quality value shall not be lower than 20. If it is lower, the sequence within the window and to its right shall be discarded; 3. After QC, the length of the read shall not be less than 75 bases; 4. The number of N bases in a single read should not exceed 5.

### Screening of differentially expressed genes (DEGs)

The DESeq2 software was used to perform differential expression analysis of genes between the two groups (We refer to this dataset as MH-S raw data in the following text.). The criteria for screening DEGs were set as *P* < 0.05 and | log2 (fold change) | >1.

### Datasets

The mRNA profiles of GSE225406 [11], GSE40885 [12], GSE121871 [13] and GSE243066 [15] were obtained from Gene Expression Omnibus (GEO, https://www.ncbi.nlm.nih.gov/geo/). GSE225406 included 3 BALF samples from mouse lung tissues and 3 BALF samples from mouse lung tissues at day 3 after intratracheal delivery of LPS determined by Illumina NovaSeq 6000 (Mus musculus), GSE121871 was generated from human AMs isolated from humanized mouse models 4 h after intratracheal inoculation with 50 mL suspensions of high-virulence or low-virulence bacterial strains, with eukaryotic transcriptome sequencing performed on the Illumina HiSeq 4000 platform, GSE243066 included mRNA sequencing data from PBMs of 24 ARDS patients and 15 healthy donors, sequenced using the Illumina NovaSeq 6000 platform, and GSE40885 included 7 BALF samples from human lung tissues and 7 BALF samples from human lung tissues after intratracheal delivery of LPS for 6 h, sequenced using the Affymetrix Human Genome U133 Plus 2.0 Array.

### Differential expression analysis

For the GSE225406 gene expression profile dataset we obtained, we used the R package DESeq2 (version 1.32.0) to perform differential analysis to obtain the DEGs between different comparison groups and the control group. Specifically, The DESeqDataSetFromMatrix function was used to construct the input matrix, further use the DESeq function for data normalization, and finally use the results function for differential analysis to ultimately obtain the differential significance of each gene. For the GSE40885 gene expression profile dataset we obtained, we used the online analysis tool GEO2R to perform differential analysis to obtain the DEGs between the experimental group and the control group. The criteria for screening DEGs were set as *P* < 0.05 and | log2 (fold change) | >1.

### Functional enrichment analysis

The Database for Annotation, Visualization and Integrated Discovery (DAVID) was employed to conduct GO and KEGG enrichment analyses. The screened significant differentially expressed genes were uploaded to the DAVID platform. With the aid of its built-in analysis tools, we aimed to identify the significantly enriched GO terms in biological processe (BP), molecular function (MF), and cellular component (CC), as well as the KEGG pathways. Based on the adjusted p-values and the number of differentially expressed genes enriched in the pathways, the top thirty significant pathways were sorted and selected (the screening criterion was a false discovery rate (FDR) < 0.05). In cases where there were fewer than 30 pathways meeting the criterion, the top-ranked pathways were selected for analysis.). Subsequently, the ggplot2 package and the goplot function in R software were utilized to visualize the results of the enrichment analysis by drawing bubble plots and circular plots.

### Analysis of core genes

We mapped all mouse genes to human homologs using biomaRt before analysis [18]. We excluded probes/genes that could not be mapped to human homologs from further analysis. We drew Venn diagrams to determine the DEGs that are commonly expressed in the three datasets. The search tool for the retrieval of interacting genes/Proteins database called STRING (https://www.string-db.org/) was used to construct the PPI network of proteins derived from shared DEGs among three datasets. STRING aims to integrate all known and predicted associations between proteins, including both physical interactions as well as functional associations. This experiment set the medium confidence score of 0.400 to generate the PPI network of common DEGs. Subsequently, we imported our PPI network into Cytoscape (v.3.10, https://cytoscape.org/) for a superior visual representation and further PPI network studies. Then, Cytohubba, a plugin in Cytoscape (https://apps.cytoscape.org/apps/cytohubba), was used to calculate the hub genes in the PPI network. Cytohubba can sequence and extract the central or target elements of a biological networks based on different network characteristics. Cytohubba has 11 methods for topological analysis from various viewpoints, and we chose Betweenness. After confirming the top 20 hub genes, the core genes were identified by comparing with the relevant gene sets in the GeneCards database.

### Analysis of common DEGs expression by quantitative Real-Time PCR (qRT-PCR)

Thirteen upregulated and one downregulated common DEGs were validated using qRT-PCR. RNA from MH-S cells was extracted (Genesand, cat#: RE716), reverse-transcribed to cDNA (Servicebio, cat#: G3329), and amplified via SYBR Green Master-Mix (Servicebio, cat#: G3326) on a Bio-rad CFX Connect system. Relative expression (stimulated vs. control) was calculated using 2^-ΔΔCT. Primers (Servicebio, Wuhan, China) and PCR amplification cycling conditions are listed in Table S1A, and S1B.

### Analysis of ACOD1 (also known as IRG1) and TNIP3 expression by Western blotting

MH-S cells were lysed with RIPA buffer containing protease/phosphatase inhibitors (Solarbio, cat#: P6730/P1260), sonicated, and centrifuged (14,000 rpm, 4 °C, 10 min). Protein concentration was determined by BCA assay (Bioworld, cat#: BD0028). Denatured samples (40 µg) were separated on 6% SDS-PAGE, transferred to PVDF membranes (Millipore Sigma, cat#: IPVH00010), blocked with QuickBlock buffer (Beyotime, cat#: P0252), and incubated overnight at 4 °C with primary antibodies. After TBST washes, membranes were incubated with HRP-conjugated secondary antibody for 1 h at RT. Bands were visualized using Immobilon substrate (Merck, cat#: WBKLS0100-1) and imaged (Amersham Imager 680, Gene Ray Electric company, USA). Quantification was performed with Image J. Antibody details are provided in the Table S1C.

### Immunofluorescence analysis of TNIP3 expression in MH-S cells

MH-S cells were fixed with 4% paraformaldehyde (GenXion, cat#: JX0100). They were then blocked for 30 min with QuickBlock™ Blocking Buffer for Immunol Staining (Beyotime, cat#: P0260), and incubated overnight with primary antibodies. After washing with PBS, they were incubated for 1 h at RT with FITC and Cy3 conjugated secondary antibody. After nuclear staining with DAPI (Servicebio, cat#G1012) and washing with PBS, the cells were checked under a confocal laser microscopy (SP8, Leica, Germany). Antibody details are provided in the Table S1C.

### Murine acute lung injury model

To avoid potential confounding effects of estrous cycle-related hormonal fluctuations in female mice and facilitate results comparison, six-to-eight-week-old SPF-grade male BALB/c mice were obtained from Guangdong Medical Experimental Animal Center (license number SCXK [YUE] 2022-0002). The mice were group-housed in pathogen-free cages at the Experimental Animal Center of Guangzhou Medical University. The housing conditions were standardized with a room temperature of 20–26 °C, humidity of 40–70%, and a 12-hour light/dark cycle. The mice had free access to water and standard rodent chow throughout the protocols. The experimental protocol was approved by the Animal Care and Use Committee of Guangzhou Medical University. After one week of acclimatization, the mice were randomly divided into two groups: the control group and ALI model group, The ALI model was induced by direct intratracheal instillation of LPS (2 mg/kg) (Escherichia coli serotype O111:B4; Sigma-Aldrich, St. Louis, MO, USA), as described in protocols from other groups [19]. At the end of the experiment, the mice were euthanized. 3% sodium pentobarbital solution was prepared with sterile physiological saline. According to the usual dose of 30 mg/kg body weight, it was converted to 1 ml/kg body weight and injected intraperitoneally. After the anesthetic depth of the mice reached an appropriate state, euthanasia was performed by cervical dislocation. Ensure that the entire process complies with animal ethics regulations and minimizes the pain of the mice as much as possible.

### Hematoxylin-eosin staining analysis

For histopathological assessment, mouse lungs were dissected and fixed in paraformaldehyde for 24 h at 4 °C. The lung tissues were then embedded in paraffin, and cut into 4-µm-thick sections. Subsequently, the sections underwent routine dewaxing using xylene I and xylene II solutions, followed by hydration with an ethanol concentration gradient. Hematoxylin and eosin staining (Servicebio, cat#: G1076) was performed to visualize nuclei and cytoplasmic structures in the sections. Bright-field images were acquired using a microscope (DM68, Leica, Germany).

### Immunohistochemistry analysis

The lung tissue sections of mice were cut into 4 μm slices after being embedded in paraffin. After antigen retrieval and washing, endogenous peroxidase activity was blocked with a 3% H_2_O_2_ solution in methanol for 20 min at room temperature in the absence of light, followed by washing with 1×PBS. The sections were then incubated with QuickBlock™ Blocking Buffer for Immunol Staining (Beyotime, cat#: P0260) for 15 min and stained overnight at 4 ℃ with a primary antibody against TNIP3. Subsequently, an HRP-conjugated goat anti-Rabbit secondary antibody was added to incubate the slices for one hour at room temperature. Next, a color reaction was developed using diaminobenzidine (Servicebio, cat#: G1212), and nuclei were counterstained with hematoxylin (Servicebio, cat#: G1004). Bright-field images were acquired using a microscope (DM68, Leica, Germany). Antibody details are provided in the Table S1C.

### Determination of the efferocytosis of MH-S cells

Jurkat T cells (obtained from ATCC) were exposed to UV light at a wavelength of 254 nm for 30 min and then returned to the incubator for 4 h to allow spontaneous apoptosis to occur, and more than 80% of the cells were observed to undergo apoptosis under the microscope using Trypan blue staining. MH-S cells treated according to the control and LPS (10 ng/mL, 72 h) groups were inoculated onto cell crawls in 12-well cell culture plates. Apoptotic Jurkat T cells were labeled with PKH26 using PKH26 Red fluorescent cell membrane staining kit (Solarbio, cat#: D0030). Apoptotic Jurkat T cells were added to each well at a ratio of 10:1 (apoptotic Jurkat T cells: MH-S cells) in 600 µl of complete culture medium and co-cultured with MH-S alveolar Macrophages for 4 h. They were then fixed with 4% paraformaldehyde, and fluorescence of unendocytosed apoptotic Jurkat T cells was quenched by incubating for 2 min with 1 ml of 0.04% Trypan Blue Stain Solution (Solarbio, cat#: C0040). MH-S cells were then blocked for 30 min with QuickBlock™ Blocking Buffer, and incubated overnight with Rabbit anti-Stab2 (diluted 1:500; Thermo Fisher Scientific, cat#: PA5-76192). After washing with PBS, they were incubated for 1 h at RT with FITC conjugated Goat Anti-Rabbit IgG (H + L) (diluted 1:200, Servicebio, cat#: GB22303). After nuclear staining with DAPI and washing with PBS, the cells were checked under a confocal laser microscope (SP8, Leica, Germany).

### Translational medicine-oriented multi-source data integration and clinical validation of core genes

Raw data from GSE121871 and GSE243066 were processed using the DESeq2 package (version 1.32.0) in R, where a count matrix was constructed using the DESeqDataSetFromMatrix function, normalized via the DESeq function, and DEGs were extracted using the results function with significance thresholds of *P* < 0.05 and | log2(fold change) |>1. Volcano plots were generated using the ggplot2 package to visualize DEG distributions. Core DEGs were validated through radar charts which were plotted using the fmsb package in R. Expression matrices from GSE243066 were subjected to Gene Set Variation Analysis (GSVA v1.48.0) using the gsva function to calculate enrichment scores for the 10-gene signature (*STAT1*, *CCL5*, *PTGS2*, *HIF1A*, *CCR5*, *ISG15*, *CD38*, *MX1*, *IRF7*, *CLEC4E*), generating a GSVA enrichment score matrix. The diagnostic performance of the GSVA enrichment score matrix was evaluated using the pROC package (v1.18.4). Receiver operating characteristic (ROC) curves were generated by initializing ROC objects with the ROC function, visualized via plot function, and quantified by calculating the AUC (area under the curve) using the auc function.

### Statistical analysis

The statistical analysis was conducted using GraphPad Prism software version 9.0.0 (GraphPad Software, San Diego, CA). Differences between two continuous groups were analysed using the independent Student’s t-test for normally distributed variables. All experiments were repeated at least three biological replicates, and the data were presented as mean ± standard error (mean ± SE). A P-value less than 0.05 was considered statistically significant.

## Results

### RNA sequencing data quality assessment of LPS-stimulated MH-S cells

To systematically characterize the LPS-induced transcriptional alterations in MH-S alveolar macrophages, we conducted RNA sequencing (RNA-seq) on cDNA libraries generated from equal quantities of total RNA isolated from untreated cells and cells exposed to LPS (10 ng/mL, 72 h). Illumina-based high-throughput sequencing produced an average of 7.0 GB raw data per group (Table S2). Following rigorous quality control, all six libraries (three biological replicates per group) exhibited optimal sequencing metrics: (1) > 90% clean reads post-adapter trimming and low-quality read removal, (2) Q30 scores exceeding 88% (base-call accuracy ≥ 99.8%), and (3) GC content of ~ 50% (Table S2). These high-quality data ensured robust downstream transcriptomic analyses.

### Cross-dataset identification of LPS-Induced differentially expressed genes

**Fig. 2:**
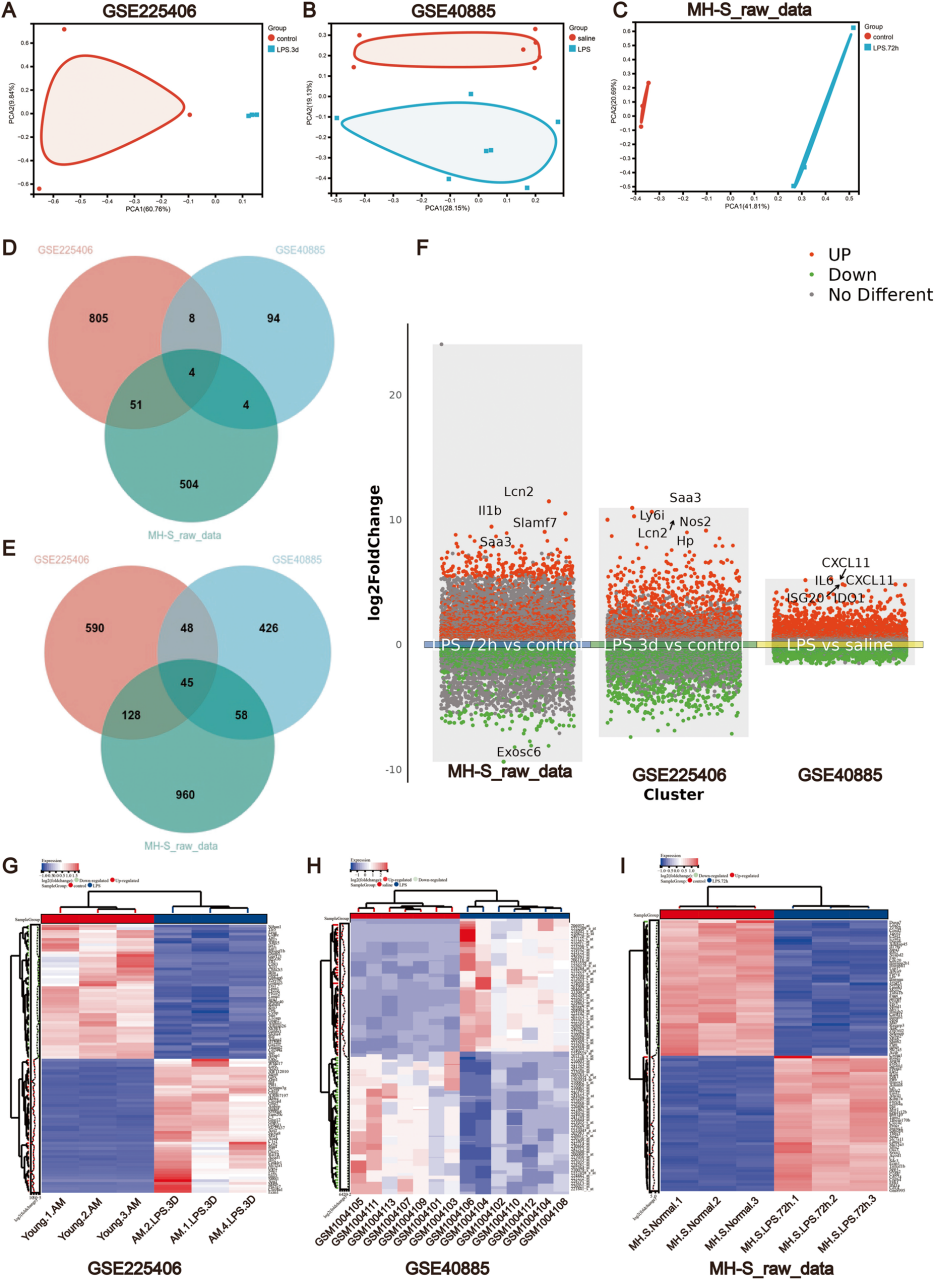
The screening of DEGs. (A - C) The principal component analysis demonstrating the separation between two groups in GSE225406 (**A)**, GSE40885 (**B)** and MH-S raw data (**C)**. (D, E) Venn diagrams identifying upregulated **(D)** and downregulated **(E)** DEGs common among the three datasets. (**F)** The volcano plots illustrating DEGs between two groups in three datasets. The red points represent upregulated genes, the green points represent downregulated genes, and the gray points denote genes without significant differential expression. (G-I) Heatmaps displaying DEGs between the LPS-treated group and the control group in GSE225406 (**G)**, GSE40885 (**H)** and MH-S raw data (**I)**. The horizontal axis represents genes, the vertical axis represents samples, red indicated high expression, blue indicate low expression

To ensure robust comparability across datasets, we first performed principal component analysis (PCA) on transcriptomic profiles from three independent sources: (1) our MH-S cell raw data (LPS vs. untreated controls), (2) the murine alveolar macrophage dataset GSE225406 (LPS vs. control), and (3) the in vivo human alveolar macrophage dataset GSE40885 (LPS vs. saline). PCA revealed clear separation between LPS-treated and control groups in all datasets (Fig. 2A-C), validating their suitability for integrative analysis. Comparative analysis identified 1,754 DEGs in our MH-S dataset (1,191 upregulated, 563 downregulated; | log2FC | >1, adjusted *p* < 0.05), 1,679 DEGs in GSE225406 (811 upregulated, 868 downregulated), and 687 DEGs in GSE40885 (577 upregulated, 110 downregulated). Intersection analysis of DEGs across all three datasets identified 45 universally upregulated genes (e.g., *ACOD1*, *CLEC4E*) and 4 consistently downregulated genes (Fig. 2D, E). These overlapping DEGs represent core transcriptional regulators of LPS-induced macrophage activation, potentially serving as key candidates for mechanistic validation. Volcano plots and hierarchical clustering heatmaps (Fig. 2F-I) visualized the global expression patterns of these DEGs, highlighting consistent LPS-driven transcriptional shifts.

### Cross-species evolutionary conservation and divergence in LPS-activated pathways of AMs

**Fig. 3:**
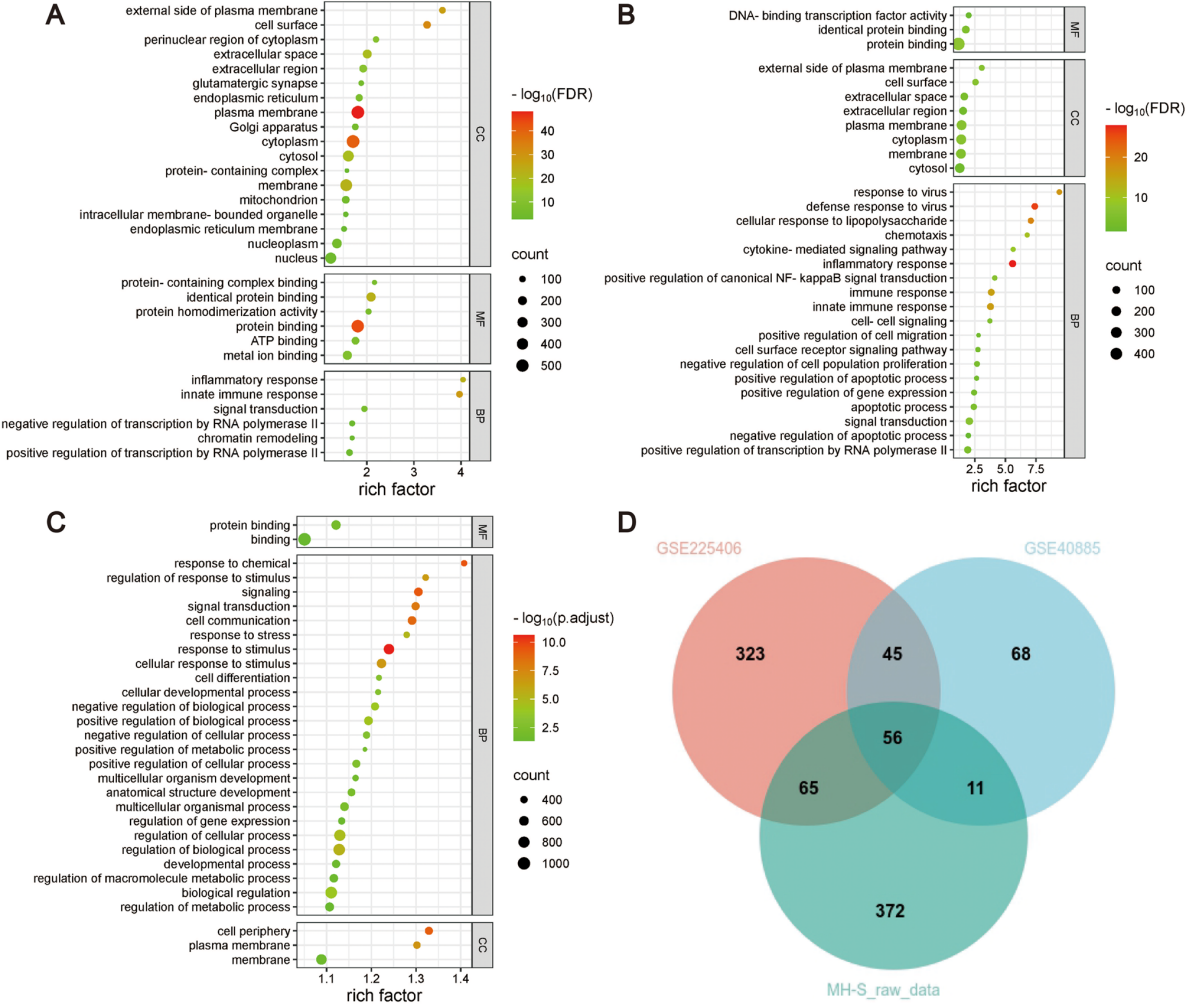
GO enrichment analysis. (A-C) Top 30 significantly enriched biological processes (BP), cellular components (CC), and molecular functions (MF) in GSE225406 (**A)**, GSE40885 (**B)**, and MH-S datasets (**C)** (adjusted *p* < 0.05). Dot size indicates the number of genes, the rich factor indicates the proportion of enriched genes for each term, and color represents the significance level. (**D)** Venn diagram showing overlapping GO terms across all three datasets

**Fig. 4:**
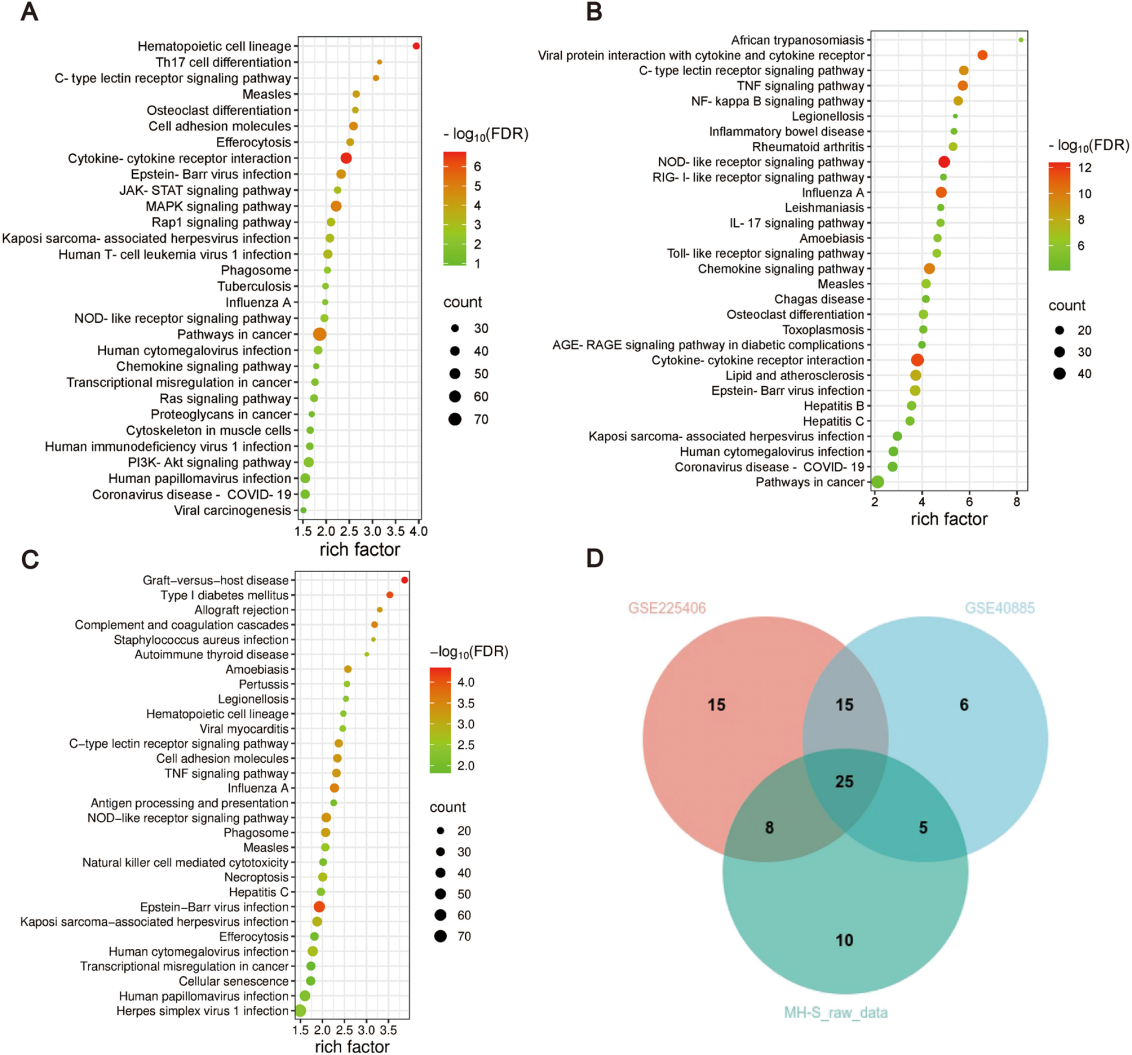
KEGG enrichment analysis. (A-C) The top 30 most significantly enriched KEGG pathways in the GSE225406 (**A)**, GSE40885 (**B)**, and MH-S raw data (**C)** datasets. Dot size indicates the number of genes involved in each pathway. The rich factor represents the proportion of enriched genes relative to the total number of genes in each pathway. Color intensity corresponds to the − log₁₀(p-value). (**D)** Venn diagram illustrating the overlap of significantly enriched KEGG pathways among the GSE225406, GSE40885, and MH-S raw data datasets

To elucidate conserved and species-specific mechanisms underlying LPS-induced AMs activation, we performed Gene Ontology (GO) and Kyoto Encyclopedia of Genes and Genomes (KEGG) pathway enrichment analyses on DEGs from three independent datasets (MH-S cells, GSE225406, and GSE40885). For each dataset, the top 30 significantly enriched terms (FDR-adjusted *p* < 0.05) were visualized, revealing distinct functional landscapes between human and murine AMs (Figs. 3A-C and 4A-C). Despite species-specific variations in biological processes (BP) and pathways, all datasets converged on immune-inflammatory networks, pathogen defense mechanisms, and cellular functional regulation.

To further explore the commonality of AMs in biological functions and signaling pathways in different datasets after LPS treatment, we performed an overlap analysis of the common GO and KEGG enrichment terms in the three datasets. Intersection analysis identified 47 conserved BP terms (e.g., “cellular response to lipopolysaccharide”, “innate immune response”, “inflammatory response”, “defense response to virus/bacterium/protozoan”, “apoptotic process” and “ERK1/ERK2 cascade”) and 25 shared KEGG pathways (e.g., “TNF signaling”, “cytokine-cytokine receptor interaction”, “pathways in cancer”, “NOD-like receptor signaling”, and “efferocytosis”) across all datasets (FDR < 0.05; Figs. 3D and 4D; Table S3). These overlapping annotations delineate a core regulatory framework for AMs during ARDS, organized into three thematic axes: (1) Innate immune activation: TLR/cytokine-driven pathogen recognition and inflammatory mediator production. (2) Signal transduction and regulatory cascades: MAPK/NF-κB cascades linking extracellular stimuli to transcriptional reprogramming. (3) Tissue repair mechanisms: Apoptosis-efferocytosis coupling to resolve inflammation and initiate repair.

To account for genomic divergence, we separately analyzed human and mouse orthologs of overlapping DEGs using species-matched annotation resources: Human AMs exhibited enriched cytoplasmic localization (CC: “cytoplasm”), 16 significantly enriched BP terms including “response to virus”, “defense response to bacterium” and “cellular response to lipopolysaccharide”, etc. (Figure S1A), and 9 significantly enriched KEGG pathways including “NOD-like receptor signaling pathway” and “Coronavirus disease - COVID-19”, etc. (Figure S1C). Murine AMs showed 2 significant CC terms including “cytoplasm” and “cytosol”, 26 significant BP terms including “defense response to virus”, “response to bacterium” and “innate immune response”, etc. (Figure S1B), and 9 significantly enriched KEGG pathways including “Hepatitis C”, “NOD-like receptor signaling pathway” and “C-type lectin receptor signaling pathway”, etc. (Figure S1D). This dual-species approach revealed evolutionary preservation of LPS-responsive immune modules while highlighting human-mouse divergence in microenvironmental adaptation.

### Core genes identification in the LPS-stimulated AMs PPI network

**Fig. 5:**
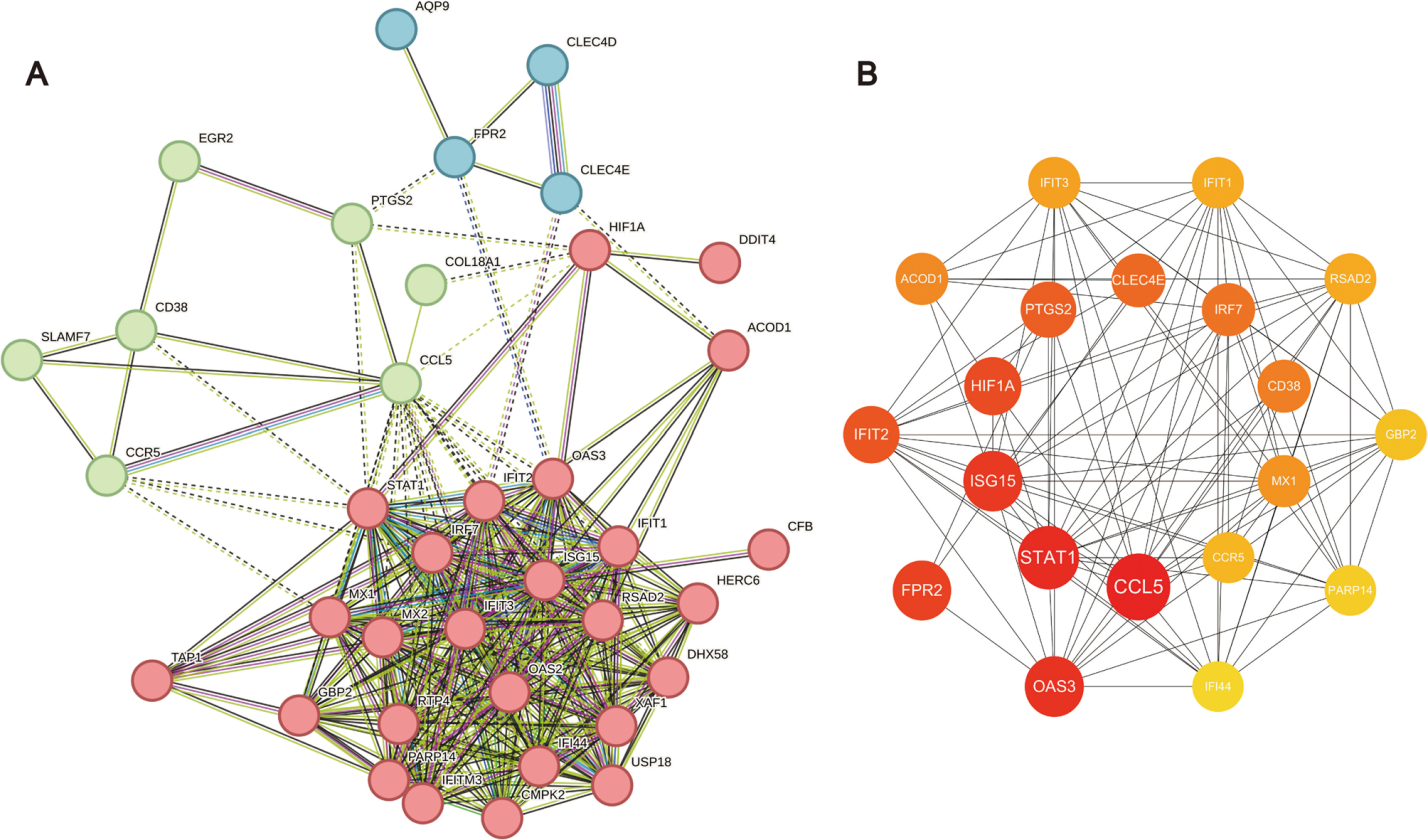
The PPI network of common DEGs was constructed using the STRING online database, followed by module analysis. (**A)** PPI network comprised 37 common DEGs. Nodes represent proteins, and edges indicate protein-protein interactions. The K-Means clustering algorithm was applied to divide the data points into three distinct clusters. (**B)** The hub genes were identified from the PPI network using the Cytohubba plug in Cytoscape. The colored nodes in the center represent the 10 core genes screened through comparative analysis with relevant gene sets in the GeneCards database

PPI network analysis was employed to elucidate the functional relationships among common DEGs. The diagram depicts a network analysis of common DEGs, where each node represents a protein with distinct levels of expression. We submitted 49 overlapping DEGs to the STRING database (v12.0, medium confidence score ≥ 0.4) to visualize their relationships as a network. To enhance the network’s clarity, we established a threshold of a minimum interaction score of 0.4 and omitted disconnected nodes. A total of 265 edges comprising the entire network were generated. The interaction network was divided into three parts according to K-means clustering, as shown in Fig. 5A. The red cluster is mainly related to interferon α/β signaling, the green cluster to interleukin-10 signaling, and the blue cluster to the innate immune response mediated by C-type lectin domain family members.

The cytoHubba tool was then used to identify the top 20 hub genes within this network, which we regarded as candidate core genes. Subsequently, we queried the GeneCards database to search for potential target genes associated with ALI pathogenesis. The search query was formulated using Boolean operators as follows: (“Alveolar macrophages” AND (“Acute Lung Injury” OR “ALI” OR “Acute Respiratory Distress Syndrome” OR “ARDS”)). This query aimed to retrieve genes that were both related to AMs and implicated in ALI/ARDS. Upon retrieving the GeneCards data, we focused on the Relevance score, a metric provided by the database to gauge the degree of association between genes and the search terms. Although the Relevance score did not have an absolute cutoff, we primarily considered genes with Relevance Scores exceeding the dataset mean (≥ 19.6) as potential candidates. The list of potential target genes obtained from GeneCards was then cross-referenced with our 20 pre-identified hub genes. After comparison and verification, 10 genes emerged as core genes common to both datasets: *STAT1*, *CCL5*, *PTGS2*, *HIF1A*, *CCR5*, *ISG15*, *CD38*, *MX1*, *IRF7*, and *CLEC4E* (Fig. 5B). These genes potentially play significant roles in the interactions between the host and bacteria.

### Validation of overlapping DEGs by qRT-PCR, Western blotting, immunohistochemical staining, and Immunofluorescence staining

**Fig. 6:**
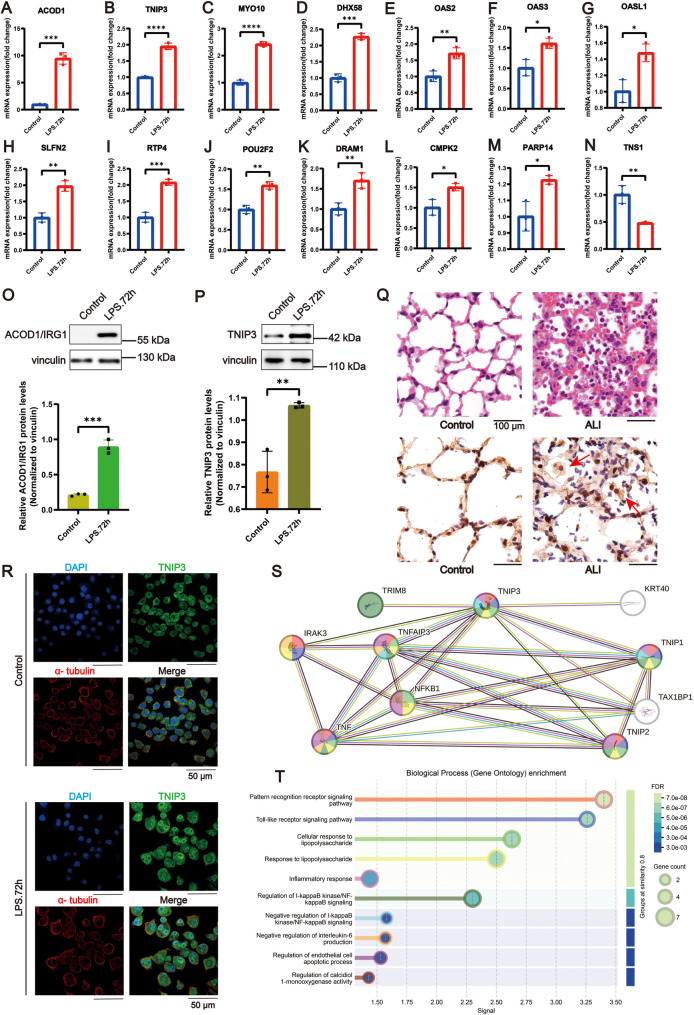
Validation of DEGs using qRT-PCR, western blot analysis, immunohistochemistry analysis and immunofluorescence analysis. (**A**-**N)** qRT - PCR was employed to assess the relative mRNA expression levels of selected common DEGs in MH-S cells after LPS treatment (10 ng/mL, 72 h). The results revealed that, in comparison to the control group, the LPS.72 h group showed a significant increase in the expression of *ACOD1*, *CMPK2*, *MYO10*, *OAS2*, *OAS3*, *PARP14*, *POU2F2*, *SLFN2*, *RTP4*, *TNIP3*, *DHX58*, *DRAM1*, *HERC6*, and *OASL1*. Conversely, *TNS1* expression was decreased (*n* = 3). (**O**, **P)** Western blot was conducted to detect the protein expression of ACOD1/IRG1 and TNIP3 in MH-S cells following treatment with 10 ng/mL LPS for 72 h. Vinculin was used as the loading control, and the ACOD1/IRG1 protein and TNIP3 protein levels were quantified and normalized to vinculin (*n* = 3). (**Q)** Representative photomicrographs of lung tissues stained with hematoxylin and eosin (H&E) were obtained, and a large inflammatory cell infiltrate was observed in the lung of ALI mice. Immunohistochemistry on adjacent paraffin sections using the anti-TNIP3 antibody. TNIP3 was mainly expressed in the nuclei and cytoplasm of AMs (as the arrows indicate). Scale bars: 100 μm. (**R**) The results of immunofluorescence staining of TNIP3 show that TNIP3 protein exhibited significant nuclear translocation in MH-S cells following LPS treatment. (**S**, **T)** Predicted functional partners of TNIP3 and the biological processes in which these genes may be involved. Data are presented as mean ± SE. *P < 0.05, **P < 0.01, ***P < 0.001 and ****P < 0.0001

To validate the common DEGs derived from bioinformatics, we assessed the relative mRNA expression levels of some common DEGs in MH-S cells treated with LPS (10 ng/mL), using qRT-PCR. The results, shown in Fig. 6A-N, revealed that the expression levels of several DEGs were significantly altered in the LPS-treated groups compared to the control groups. Specifically, the expression levels of *ACOD1*, *CMPK2*, *MYO10*, *OAS2*, *OAS3*, *PARP14*, *POU2F2*, *SLFN2*, *RTP4*, *TNIP3*, *DHX58*, *DRAM1*, *HERC6*, *and OASL1* were significantly upregulated, whereas the expression level of TNS1 was downregulated in the LPS-treated groups compared to the control groups. Western blot analysis demonstrated that ACOD1 and TNIP3 protein were dramatically upregulated in MH-S cells following LPS stimulation for 72 h (Fig. 6O, P). Furthermore, we performed initial qualitative and spatial assessments of TNIP3 expression in mouse lung tissue and MH-S cells using immunohistochemistry and immunofluorescence. In adult mouse lungs, TNIP3 immunoreactivity was primarily localized within both the nuclei and cytoplasm of AMs. Notably, lung tissues from ALI models displayed significantly elevated TNIP3 expression, with intensified staining intensity and broader cellular distribution patterns, particularly in AMs-rich regions (Fig. 6Q). Moreover, we discovered that long-term LPS stimulation (72 h) induced significant nuclear translocation of TNIP3 protein in MH-S cells, revealing the dynamic localization changes of this protein. This finding suggests that TNIP3 might participate in the precise regulation of immune responses by targeting inflammation-related gene expression in the nucleus or interacting with nuclear signaling molecules (Fig. 6R). We preliminarily predicted proteins potentially interacting with TNIP3 using the STRING database, including TNFAIP3, NF-κB, and TNIP1 (Fig. 6S). These genes are significantly enriched in biological processes such as the “pattern recognition receptor signaling pathway”, “response to lipopolysaccharide”, “regulation of I-kappaB kinase/NF-kappaB signaling”, and “Toll-like receptor signaling pathway” (Fig. 6T), which suggest that TNIP3 may play a potential regulatory role in AMs activation during the progression of ALI/ARDS. Collectively, these findings corroborate the bioinformatics analysis results, thereby validating the DEGs identified in our study.

### LPS treatment reduces efferocytosis capacity in MH-S cells

**Fig. 7:**
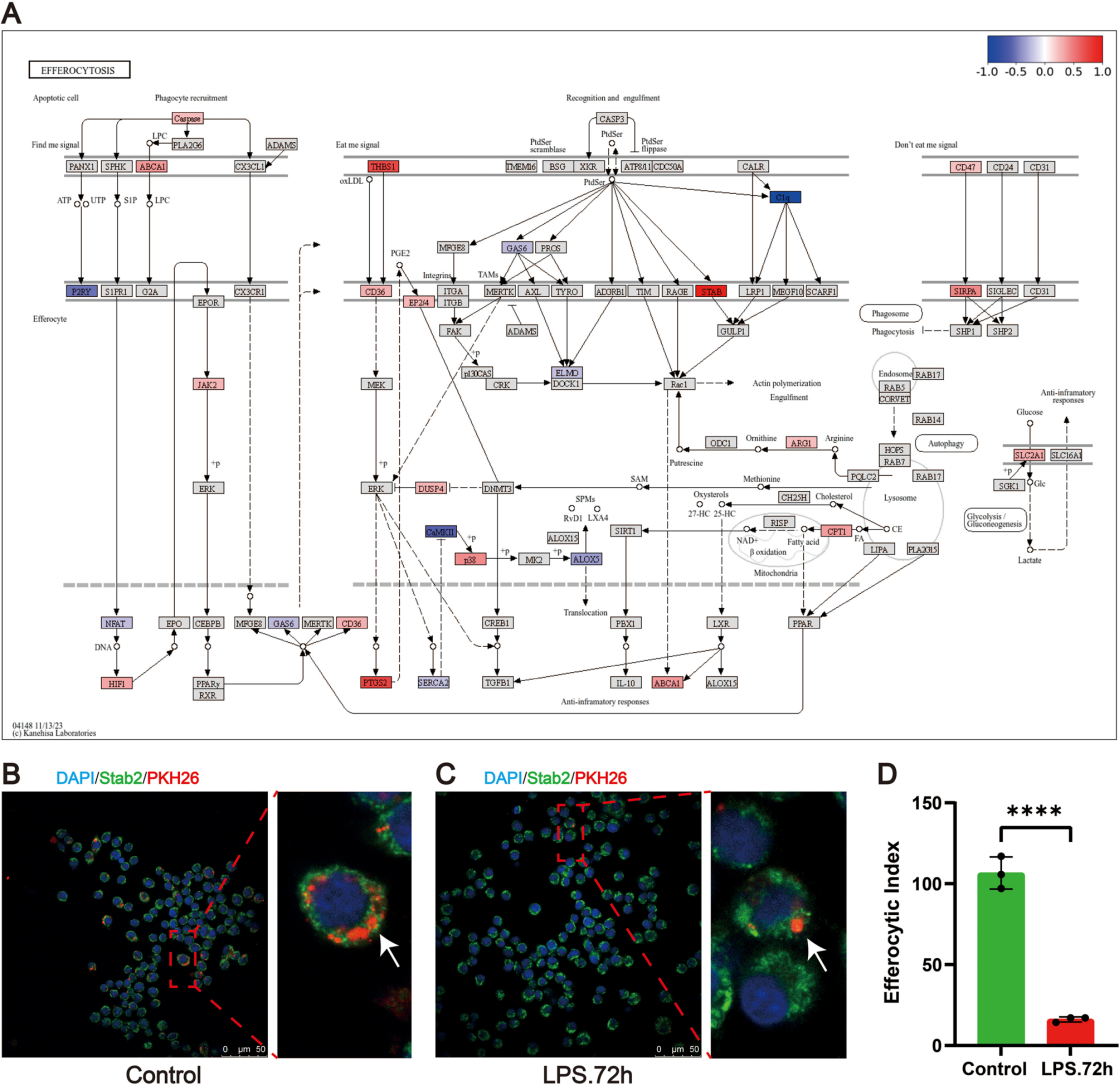
LPS inhibits the phagocytosis of apoptotic cells by MH-S cells. (**A)** The core genes identified in the “Efferocytosis” process were derived from KEGG enrichment analysis of the MH-S raw data dataset. Red indicates high gene expression levels, while blue represents low expression levels. (**B)** Stab2-positive MH-S cells that had engulfed apoptotic cytoplasmic debris were observed after co-culture with apoptotic cells for 4 h. Confocal microscopy with z-sectioning showed some apoptotic cytoplasmic debris are surrounded by a higher concentration of Stab2 staining than surrounding plasma membrane (as indicated by the arrow). (**C)** LPS-treated MH-S cells exhibited significantly reduced engulfment of apoptotic cells. The scale bar is 50 μm. All laser confocal microscope photos were taken using a 40× objective lens. (**D)** Efferocytosis index of MH-S cells as observed under confocal microscopy. The results are presented as Mean ± SE. *N* = 3, **** *P* < 0.0001

The “Efferocytosis” pathway was significantly enriched in all three datasets, and through mining of the results of KEGG enrichment analysis, genes with significant differential expression in this pathway were identified, including *Stab2*, *Abca1*, *Cd36*, *Ptgs2*, *Elmo1*, etc. (Fig. 7A), prompting us to investigate whether LPS treatment affects the efferocytosis function of AMs. This analysis aimed to explore potential changes in the ability of AMs to clear apoptotic cells in the pulmonary inflammatory environment. To this end, we used an anti-Stab2 antibody for cytoplasmic staining to preliminarily assess whether this phosphatidylserine receptor (one of the key efferocytosis receptors) mediates the efferocytosis function of AMs.

MH-S cells were treated with LPS (10 ng/mL) for 72 h and subsequently co-cultured with PKH26-labeled apoptotic Jurkat T cells to determine efferocytosis. Confocal microscopy with z-sectioning revealed that debris (identified by PKH26 staining) was localized within the cytoplasm of Stab2-positive macrophages. A single confocal slice demonstrated that some apoptotic cytoplasmic debris was surrounded by higher concentrations of Stab2 staining compared to the surrounding plasma membrane (Fig. 7B-C). Compared with the control group, LPS treatment significantly reduced the efferocytosis index by 85%, as assessed by immunofluorescence analysis (Fig. 7D), suggesting a potential impairment of inflammation resolution in ALI/ARDS pathogenesis. However, whether this defect is a cause or consequence of ARDS requires further mechanistic validation.

### Integrated analysis of multi-source data reveals cross-species conservation and diagnostic potential of core genes in ARDS

**Fig. 8:**
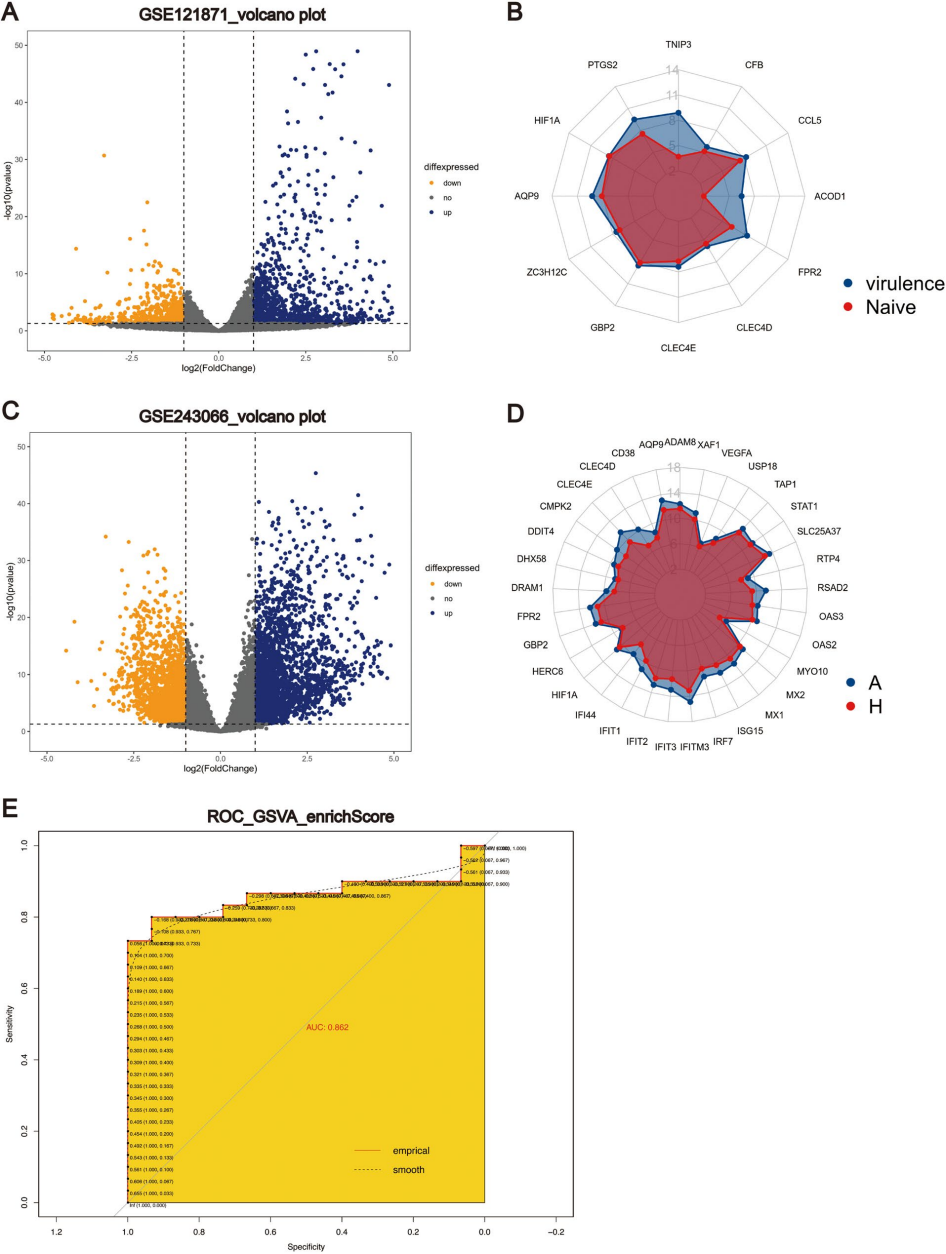
The screening of DEGs in GSE121871 and GSE243066, and the cross-species conservation of core genes and the exploration of their clinical diagnostic efficacy. (A, B) The volcano plot (**A)** and radar chart (**B)** illustrating DEGs in GSE121871 dataset. The blue points represent upregulated genes, the yellow points represent downregulated genes, and the gray points denote genes without significant differential expression. The end points of the rays are labeled with different gene names. The blue area represents the gene expression level of the “virulence” group, and the red area represents the gene expression level of the “Naive” group. (C, D) The volcano plot (**C)** and radar chart (**D)** illustrating DEGs in GSE243066 dataset. The blue area represents the gene expression level of the “ARDS (A)” group, and the red area represents the gene expression level of the “Healthy (H)” group. (**E)** ROC curves of the core gene set GSVA enrich score in the GSE243066 dataset

Subsequently, by integrating GSE121871 and GSE243066 datasets, we systematically validated the conservation and clinical diagnostic potential of the 10 core genes (*STAT1*, *CCL5*, *PTGS2*, *HIF1A*, *CCR5*, *ISG15*, *CD38*, *MX1*, *IRF7*, *CLEC4E*) identified in previous cross-species and cross-pathological model analyses. The GSE121871 dataset, established on a humanized mouse model, mimicked bacterial pneumonia induced by *Pseudomonas aeruginosa* infection. Through differential expression analysis between the high-virulence strain group and the control group, we identified 732 upregulated genes and 354 downregulated genes (Fig. 8A). Among these DEGs, 12 genes overlapped with the common upregulated genes we had identified (Fig. 8B), including four core genes (*CCL5*, *PTGS2*, *HIF1A*, *and CLEC4E*). This observation further supports the critical role of these genes in disease progression.

The GSE243066 dataset was utilized to validate the systemic expression patterns of the core genes from the perspective of clinical samples. Although this dataset was derived from PBMs of ARDS patients rather than AMs, its systemic inflammatory characteristics closely correlated with clinical phenotypes of ARDS, such as cytokine storms and multi-organ dysfunction. Differential expression analysis between the ARDS group and the control group identified 2,552 upregulated genes and 2,111 downregulated genes (Fig. 8C). Among these, 33 genes overlapped with our common upregulated gene set (Fig. 8D), including seven core genes (*CLEC4E*, *IRF7*, *HIF1A*, *ISG15*, *MX1*, *CD38*, *STAT1*).

To further assess the diagnostic value, we performed a Gene Set Variation Analysis (GSVA) to calculate the enrichment scores of the core gene set across groups in GSE243066. The results showed significantly higher GSVA enrichment scores in the ARDS group compared to the control group. ROC curve analysis further confirmed the efficacy of these genes in distinguishing ARDS patients from healthy individuals (AUC = 0.86) (Fig. 8E). These findings suggest that the core gene set not only exhibits cross-species conservation but also holds potential as clinical biomarkers for diagnosis of ARDS, but warranting validation in alveolar macrophage samples from ARDS patients.

## Discussion

In this study, we aimed to elucidate the transcriptional and functional changes in AMs during ALI/ARDS by integrating in vitro and cross-species transcriptomic analyses. Using an LPS-induced MH-S cell model and publicly available murine (GSE225406) and human (GSE40885) datasets, we identified 45 conserved upregulated genes and 4 downregulated genes across species, highlighting core transcriptional regulators of LPS-induced macrophage activation. Functional enrichment analysis revealed significant involvement of immune-inflammatory pathways, including “TNF signaling,” “Cytokine-cytokine receptor interaction,” and “NOD-like receptor signaling.” Additionally, we identified 10 core genes (e.g., *CLEC4E*, *IRF7*, *ISG15*) potentially central to AM-mediated ALI/ARDS pathogenesis. Experimental validation confirmed the upregulation of key genes (e.g., *ACOD1*, *TNIP3*) and demonstrated that LPS treatment significantly impaired the efferocytosis capacity of AMs, a critical function for resolving inflammation. This study is the first to integrate cross-species AM transcriptomics to identify conserved inflammatory modules, and provides preliminary evidence suggesting a potential association between efferocytosis impairment and Stab2 dysregulation in ARDS.

Our findings provide novel insights into the molecular mechanisms underlying AM-mediated ALI/ARDS. The conserved upregulation of genes such as *STAT1*, *CCL5*, *PTGS2*, and *HIF1A* aligns with previous studies demonstrating their roles in ALI/ARDS pathogenesis. For instance, in LPS-induced ALI animal models, both JAK2 and STAT1 were found activated in lung tissues [20]; STAT1 activation mediates LPS-induced lethality in murine models [21], while elevated CCL5 levels correlate with ARDS severity and serve as an independent risk factor for lung injury progression [22]. *PTGS2* (also known as *COX2*), a rate-limiting enzyme in prostaglandin (PGs) synthesis, is induced to be highly expressed in the early stage of ALI [23]. It catalyzes the conversion of arachidonic acid into pro-inflammatory prostaglandins (such as PGE₂). These PGs can induce neutrophil chemotaxis, pulmonary microvascular constriction, and increased vascular permeability, thereby exacerbating alveolar edema and tissue damage [24]. HIF1A, known for its protective effects during ALI, stabilizes HIF-dependent glucose metabolism in alveolar epithelial cells, forming an endogenous feedback loop to mitigate pulmonary inflammation [25]. In obesity, reduced ACOD1 expression in AMs worsens LPS-induced lung injury, possibly due to increased GFI1, a transcriptional repressor. This suggests the GFI1-ACOD1 pathway as a target for treating obesity-related lung issues [26]. Notably, our study observed significant ACOD1 upregulation across all three datasets, warranting further investigation into its protective role and molecular mechanisms in ALI/ARDS.

Moreover, we validated the upregulation of TNIP3 and, for the first time, discovered that long-term LPS stimulation (72 h) induces significant nuclear translocation of TNIP3 protein in MH-S cells. This discovery expands the understanding of TNIP3’s role in inflammation regulation: previous studies have primarily focused on TNIP3’s cytoplasmic functions in suppressing classical inflammatory pathways such as NF-κB, for example, by inhibiting the activation of TRAF6 and IKKβ downstream of TLR4 to block NF-κB-dependent expression of inflammatory cytokines (IL-6, TNF-α) [27], as well as by directly binding to the cytoplasmic kinase TGF-β-activated kinase 1 (TAK1) to suppress its ubiquitination and subsequent activation of NF-κB and JNK pathways [28]. To date, there are no research reports on the role of this gene in the pathophysiological process of ALI/ARDS. These findings suggest that TNIP3 may regulate inflammatory gene expression through nuclear localization, a hypothesis that requires further mechanistic studies to validate. In the future, ChIP-seq experiments will be required to identify the target genes of TNIP3 in AMs and clarify its nuclear regulatory network.

Functional enrichment analysis on DEGs from each dataset and the shared DEGs revealed significant enrichment of GO terms in biological processes, including immune-inflammatory responses, cellular signaling transduction, cell migration and apoptosis, and antiviral defense mechanisms. KEGG pathway analysis further highlighted inflammation/infection-related pathways (e.g., “Cytokine-cytokine receptor interaction”, “TNF signaling”, and “Toll-like/NOD-like receptor signaling”) and cancer-associated pathways. Consistent with established mechanisms [29, 30], LPS activated classical inflammatory pathways in AMs. The enrichment of “inflammatory response” and “Toll-like/NOD-like receptor signaling” aligns with previous findings. During the acute phase of ARDS, exogenous pathogen-associated molecular patterns (PAMPs) or endogenous damage-associated molecular patterns (DAMPs) in the alveoli activate AMs through Toll-like receptors (TLRs) and nucleotide-binding oligomerization domain-like receptors (NLRs) [31, 32]. Moreover, TLR4 signaling may activate IRF3 via TRIF-dependent pathways to induce IFN-β production and subsequent JAK-STAT activation [21]. In the recovery stage of ARDS, AMs transition to a reparative phenotype, secreting anti-inflammatory cytokines (IL-10, TGF-β) to resolve inflammation and promote tissue repair [31, 33]. Our observed enrichment of “negative regulation of inflammatory response” and “efferocytosis” pathways supports their dual roles in inflammation initiation and resolution. However, mechanistic insights into macrophage regulation during the resolution phase remain limited, particularly regarding efferocytosis-related gene dynamics.

The enrichment of antiviral pathways (“defense response to virus,” “cytosolic DNA-sensing”) suggests that LPS-induced interferon signaling may enhance macrophage antiviral capacity. LPS-induced pro-inflammatory cytokines (TNF-α, IL-6) upregulate interferon-stimulated genes (ISGs), indirectly enhancing antiviral effector production [34, 35]. The cross-activation of innate immune pathways may explain the concurrent activation of antiviral immune response-related pathways in AMs. Cancer pathway enrichment (“Pathways in cancer”) underscores molecular links between inflammation and oncogenesis. Key genes (TNF, NF-κB1, VEGFA, STAT1) participate in both inflammatory and carcinogenic processes [20, 36, 37].

To further explore the functional relationships among common DEGs, we constructed a PPI network and applied K-means clustering to categorize the nodes into three distinct clusters. The red cluster is related to “Interferon Alpha/Beta Signaling”, suggesting that although LPS itself is a bacterial product, the interferon signal it induces may enhance the host’s defense against potential viral infections through cross-reactivity. The green cluster is related to “Interleukin-10 Signaling”. IL-10 is a classic anti-inflammatory cytokine that induces STAT3 phosphorylation and entry into the nucleus by activating the JAK1-TYK2-STAT3 signaling pathway, selectively inhibiting the transcription of pro-inflammatory genes (such as TNF-α, IL-6) [38, 39]. This pathway establishes a dynamic equilibrium with the pro-inflammatory role of AMs during the early stages of ALI/ARDS, reflecting the self-regulatory nature of immune responses. The blue cluster is associated with “C-type lectin domain family 12 member A/B (Clec12a/B) and Chromoblastomycosis”. Clec12a, a member of the myeloid C-type lectin receptor (CLRs) family, suppresses inflammatory responses by negatively regulating the activation of granulocytes and monocytes [40]. By cross-referencing with relevant gene sets in the GeneCards database, we identified 10 core genes potentially involved in the pathophysiological processes of ALI/ARDS influenced by AMs: *STAT1*, *CCL5*, *PTGS2*, *HIF1A*, *CCR5*, *ISG15*, *CD38*, *MX1*, *IRF7*, and *CLEC4E*. Reduced CD38 expression suppresses LPS-induced inflammation, underscoring its role as a pro-inflammatory regulator [41]. IRF7 promotes ferroptosis and M1 macrophage polarization via NF-κB signaling in sepsis-induced ALI, intensifying inflammatory responses and lung injury [42]. CLEC4E, a pattern recognition receptor (PRR), synergistically activates macrophage autophagy with TLR4 during Mycobacterium tuberculosis infection, suppressing bacterial survival [43]. However, its role in regulating AMs’ inflammatory responses in ALI/ARDS remains unexplored.

Beyond the LPS-induced bacterial model explored here, our identification of conserved core genes in AMs holds potential relevance for ARDS of diverse etiologies, particularly viral infections. Notably, several genes within our signature, such as ISG15 and MX1, are classical ISGs with well-established roles in antiviral defense [34, 44]. Their prominent upregulation across our integrated datasets suggests a conserved AM response module that may be activated not only by bacterial PAMPs like LPS but also by viral nucleic acids sensed via pattern recognition receptors. This is highly pertinent to viral ARDS, including severe COVID-19, where dysregulated interferon signaling and hyperinflammation are hallmarks [45]. For instance, ISG15, an interferon-stimulated gene, is massively released by macrophages during SARS-CoV-2 infection, amplifying pro-inflammatory cytokine and chemokine secretion and potentially contributing to hyperinflammatory responses in COVID-19 [44]. Similarly, MX1 is a key response protein in SARS-CoV-2 infection, and its expression increases significantly with each unit increase in viral load [46]. While our study primarily focused on bacterial triggers and utilized LPS models, the strong representation of antiviral ISGs within the conserved core signature implies its broader applicability. Future investigations should explicitly validate this gene set in models of viral pneumonia like influenza or SARS-CoV-2 and in clinical samples from patients with non-bacterial ARDS to confirm its utility as a pan-etiology diagnostic or therapeutic target panel.

Notably, all datasets showed “Efferocytosis” pathway enrichment, reflecting AMs’ critical phagocytic function [47]. The evaluation of BALF in patients with sepsis-associated ALI reveals that there is impaired phagocytic function of AMs and increased neutrophil apoptosis in ALI patients, and there is a negative correlation trend between AMs proliferation and neutrophil apoptosis, which has a negative impact on the mortality rate of ALI [48], suggesting that upregulating AMs’ phagocytosis of apoptotic neutrophils is a valuable strategy for improving ALI. Additionally, relevant evidence indicates that there is impaired efferocytosis of phagocytes in most lung diseases [49]. Our experimental results also show that the efferocytosis of AMs is reduced after exposure to LPS, and stabilin-2 may be one of the efferocytosis receptors mediating contact with apoptotic cells.

Finally, we validated the cross-species conservation and diagnostic potential of a set of core genes (including *CLEC4E*, *IRF7*, *ISG15*, etc.) in both pre-clinical models and clinical samples of ARDS. The GSE121871 dataset, which was based on a humanized mouse model mimicking bacterial pneumonia caused by Pseudomonas aeruginosa infection, offered valuable insights. The inflammatory mechanisms observed in this dataset, such as the activation of the TLR4/NF-κB pathway, complemented the original LPS model. Differential expression analysis in this dataset showed a significant upregulation of four core genes (*CCL5*, *PTGS2*, *HIF1A*, and *CLEC4E*) in the infection group. This finding strongly indicates the conserved roles of these genes in pathogen-driven inflammatory responses across species, further supporting their potential as key players in the disease progression of ARDS. Although GSE243066 was based on PBMs, differential analysis identified seven overlapping core genes (*CLEC4E*, *IRF7*, *STAT1*, etc.), and GSVA demonstrated that their combination effectively distinguished ARDS patients from healthy individuals, as evidenced by AUC of 0.86. This suggests that these genes may indirectly reflect pulmonary pathological processes through systemic inflammatory features. However, the heterogeneity of PBMs and the lack of AMs data from BALF limited the direct clinical relevance of our findings. AMs are directly involved in local inflammatory responses within the alveolar microenvironment, while PBMs circulate systemically and are influenced by systemic inflammation and compensatory mechanisms [16]. As a result, the transcriptomes of AMs and PBMs show significant divergence due to their distinct cellular localization and functions. Peripheral blood signals may not accurately reflect lung-specific immune responses, which could lead to an incomplete understanding of the role of core genes in the local lung microenvironment. To address these limitations, future studies should focus on directly analyzing the transcriptomes or proteomes of AMs from BALF of ARDS patients.

As the direct site of pathological changes, AMs obtained via bronchoalveolar lavage (BAL) represent an ideal sample source for validating and applying these core genes. Measuring the gene expression of AMs in BALF can most accurately reflect the local inflammatory microenvironment driving ARDS. However, BAL is invasive, which limits its routine use. PBMs serve as a less invasive and more readily accessible alternative sample. We found that the core gene signature retains discriminative capacity in PBMs despite their reflection of systemic conditions; this is promising for potential point-of-care testing applications. While PBM-based signatures may be less specific than those from AMs in BALF for solely assessing lung injury, they can function as valuable screening tools or prognostic indicators.

Our research has some limitations. First, our predictions are based on bioinformatics analysis; functional validation in animal models and clinical samples is needed to confirm their causal roles in ARDS, so further experimental verification is required. Second, we did not consider the factor of time variation, while alveolar Macrophage genes have a dynamic change pattern. Third, currently, no appropriate clinical data have been found to verify the possibility of using the gene set including these 10 core genes as a biomarker for diagnosing ALI/ARDS, and direct validation in alveolar macrophages from ARDS patients is necessary to confirm clinical relevance.

## Conclusion

This integrative analysis uncovers conserved LPS-induced inflammatory modules in AMs, and species-specific adaptations. Core genes (e.g., *CLEC4E*, *IRF7*) and efferocytosis pathways emerge as critical regulators, and the ARDS AM-related GSVA enrichment score may be helpful for the diagnosis of ARDS. These findings advance our understanding of ALI/ARDS pathogenesis and provide a translational framework for developing targeted therapies. However, further experimental validation is necessary to confirm the functional roles of the identified DEGs. Future studies should validate these findings in preclinical models and clinical trials to explore their translational potential in ALI/ARDS.

## Supplementary Information

Supplementary material 1.

Supplementary material 2.

Supplementary material 3.

## Data Availability

All relevant data and materials supporting the conclusions of this article are available in the GEO database, [https://www.ncbi.nlm.nih.gov/geo/](https:/www.ncbi.nlm.nih.gov/geo). The eukaryotic transcriptome sequencing data of MH-S cells presented in this study are publicly available in the NCBI SRA repository under project accession number PRJNA1247882, accessible at: [https://www.ncbi.nlm.nih.gov/sra/?term=PRJNA1247882](https:/www.ncbi.nlm.nih.gov/sra/?term=PRJNA1247882). For additional information, please contact 2021111305@stu.gzhmu.edu.cn.

## Abbreviations

ALI: acute lung injury
ARDS: acute respiratory distress syndrome
AMs: alveolar macrophages
LPS: lipopolysaccharide
DEGs: differentially expressed genes
GO: Gene Ontology
KEGG: Kyoto Encyclopedia of Genes and Genomes
PPI: protein-protein interaction
Stab2: stabilin-2
ICUs: intensive care units
IL: interleukins
TNF: tumor necrosis factor
BALF: bronchoalveolar lavage fluid
VAP: ventilator-associated pneumonia
PBMs: peripheral blood monocytes
GSVA: gene set variation analysis
QC: quality control
DAVID: Database for Annotation, Visualization and Integrated Discovery
BP: biological process
MF: molecular function
CC: cellular component
FDR: false discovery rate
qRT-PCR: quantitative real-time PCR
ROC: receiver operating characteristic
AUC: area under the curve
PAMPs: pathogen-associated molecular patterns
DAMPs: endogenous damage-associated molecular patterns
TLRs: Toll-like receptors
NLRs: nucleotide-binding oligomerization domain-like receptors
ISGs: interferon-stimulated genes
CLRs: C-type lectin receptors
PRR: pattern recognition receptor
