# Supplementary material for: Integrative transcriptomic analysis reveals cross-species conserved core genes and pathways in alveolar macrophages during ALI/ARDS
Source: BMC Pulm Med. 2025 Oct 2;25:447. doi: 10.1186/s12890-025-03928-y (PMC12492643; doi:10.1186/s12890-025-03928-y)
Supplement: Supplementary file 1 — Supplementary material 1. [file 12890_2025_3928_MOESM1_ESM.docx]

**A**

| Gene | Forward Sequences (5’-3’) | Reverse Sequences (5’-3’) |
| --- | --- | --- |
| ACOD1 | TCTACAGTTCCAACACCTCCAGC | TCGTCCCTGTACTTCAATACCAAC |
| CMPK2 | AGGAGGTCCAGAAAGGGAAGTT | AAAAGGCTCTTCGAATGATAGTAGG |
| MYO10 | GTCAGGTCCACTCGTCCACAGA | AGAATCAATCAGCCCGACATCC |
| OAS3 | TGGGGTCGCTAAACATCACC | AGGGTCTCTGAGTGACAGGT |
| PARP14 | GAGAAAGTATCCGAGATGGTGC | GGTGGCTGATACAGTCAACAATAA |
| POU2F2 | GAACAGTTTGCTCGCACCTTC | TCTGCGTCGTTGAGCCACTT |
| CLEC4N | TGGACCAGCCCAGTAGAAGAC | TCCGAAAGACCCAGGAAGTAA |
| SLFN2 | GCAACTGAGCAAAGCAACCA | GCTCCGAGATTTAGACCCAGC |
| RTP4 | AAGTGGAGCCTGCATTTGGATA | GGAACCTGCCAAGCACTGTCT |
| TNIP3 | AATCAACAATGGGACCAGCAA | GCCCTCTGTCACACTTTGCTT |
| DHX58 | TGGTGGTACTGGTCAATAGGGT | GCTCTACGTGTTCATCCTCCTCA |
| DRAM1 | GATTGCCTGTGCTTCACTCATT | ACACTCTGGAAATCTTGGATGAATG |
| OASL1 | ACTGGACCAAGCACTACACG | CACGGTCACCTGGATATCGG |
| OAS2 | GGTACCCTTGTTCTCTTCATGGAC | GGGATTCTCATTGGAGCGTAG |
| TNS1 | GGACCTGGTGTATGTCACCG | CAGCCGAATTCCAGGACCTT |
| GAPDH | CCTCGTCCCGTAGACAAAATG | TGAGGTCAATGAAGGGGTCGT |

**B**

| Stage1 | Stage2（40 cycles） | Stage3（Melt Curve） |
| --- | --- | --- |
| 95℃， 30s  Pre-denaturation | 95℃， 15s Denaturation  60℃， 30s Anealing/Extension | 65℃→95℃ |

**C**

| Primary Antibody Name | Primary Antibody Product Number | Primary Antibody Manufacturer | Primary antibody species | Primary Antibody Dilution Ratio | Corresponding secondary antibody name |
| --- | --- | --- | --- | --- | --- |
| Stabilin-2 (Stab2) (IF) | PA5-76192 | Thermo Fisher Scientific | rab | 1:500 | FITC conjugated Goat Anti-Rabbit IgG (H+L) (diluted 1:200, Servicebio, cat#: GB22303) |
| ACOD1/IRG1 (WB) | ab222411 | abcam | rab | 1:1000 | Goat anti-rabbit IgG (H+P) HRP  (diluted 1:5000, Bioss, cat#: bs-0295G-HRP) |
| TNIP3 (WB) | PA5-103506 | Thermo Fisher Scientific | rab | 1:1000 | Goat anti-rabbit IgG (H+P) HRP  (diluted 1:5000, Bioss, cat#: bs-0295G-HRP) |
| TNIP3 (IHC) | PA5-103506 | Thermo Fisher Scientific | rab | 1:200 | Goat anti-rabbit IgG (H+P) HRP  (diluted 1:400, Servicebio, cat#: GB23303) |
| TNIP3 (IF) | PA5-103506 | Thermo Fisher Scientific | rab | 1:200 | FITC conjugated Goat Anti-Rabbit IgG (H+L) (diluted 1:200, Servicebio, cat#: GB22303) |
| Vinculin (WB) | 26520-1-AP | Proteintech | rab | 1:10000 | Goat anti-rabbit IgG (H+P) HRP  (diluted 1:5000, Bioss, cat#: bs-0295G-HRP) |
| α-tubulin (IF) | GB15200 | Servicebio | Mus | 1:1000 | Cy3 conjugated Goat Anti-Mouse IgG (H+L) (diluted 1:500, Servicebio, cat#: GB21301) |

**Table S1. Primers (A) and PCR amplification cycling conditions (B) used in real-time polymerase chain reaction, and the information of the antibodies used (C).**

| Sample | Raw reads | Clean reads | Clean bases(G) | Clean bases ratio(%) | Q30(%) | GC content(%) |
| --- | --- | --- | --- | --- | --- | --- |
| MH.S.Normal.1 | 44831642 | 40724922 | 6.09 | 90.62 | 94.48 | 51.71 |
| MH.S.Normal.2 | 42478902 | 39911378 | 5.96 | 93.56 | 94.38 | 51.41 |
| MH.S.Normal.3 | 45865850 | 43390014 | 6.49 | 94.33 | 93.46 | 51.43 |
| MH.S.LPS.72h.1 | 62549018 | 57884274 | 8.66 | 92.32 | 88.33 | 49.72 |
| MH.S.LPS.72h.2 | 54642140 | 51010558 | 7.63 | 93.05 | 92.15 | 49.88 |
| MH.S.LPS.72h.3 | 52169592 | 48085828 | 7.19 | 91.83 | 91.66 | 49.76 |

**Table S2. Quality analysis results of sequencing data**

MH.S.Normal.1, 2, and 3 were control cell samples and MH.S.LPS.72h.1, 2, and 3 were LPS-treated cell samples. Q30: Phred quality score 30; GC: Guanine and cytosine.

| **Category** | **Term** | **name** |
| --- | --- | --- |
| GOTERM_CC_DIRECT | GO:0005886 | plasma membrane |
| GOTERM_MF_DIRECT | GO:0005515 | protein binding |
| GOTERM_CC_DIRECT | GO:0016020 | membrane |
| GOTERM_MF_DIRECT | GO:0042802 | identical protein binding |
| GOTERM_CC_DIRECT | GO:0005615 | extracellular space |
| GOTERM_CC_DIRECT | GO:0005576 | extracellular region |
| GOTERM_CC_DIRECT | GO:0009986 | cell surface |
| GOTERM_CC_DIRECT | GO:0009897 | external side of plasma membrane |
| GOTERM_BP_DIRECT | GO:0045087 | innate immune response |
| GOTERM_BP_DIRECT | GO:0007165 | signal transduction |
| GOTERM_BP_DIRECT | GO:0006954 | inflammatory response |
| GOTERM_BP_DIRECT | GO:0006955 | immune response |
| GOTERM_BP_DIRECT | GO:0006915 | apoptotic process |
| GOTERM_BP_DIRECT | GO:0051607 | defense response to virus |
| GOTERM_BP_DIRECT | GO:0043066 | negative regulation of apoptotic process |
| GOTERM_BP_DIRECT | GO:0010628 | positive regulation of gene expression |
| GOTERM_BP_DIRECT | GO:0001525 | angiogenesis |
| GOTERM_BP_DIRECT | GO:0030335 | positive regulation of cell migration |
| GOTERM_BP_DIRECT | GO:0043065 | positive regulation of apoptotic process |
| GOTERM_BP_DIRECT | GO:0071222 | cellular response to lipopolysaccharide |
| GOTERM_BP_DIRECT | GO:0007166 | cell surface receptor signaling pathway |
| GOTERM_BP_DIRECT | GO:0042742 | defense response to bacterium |
| GOTERM_BP_DIRECT | GO:0032496 | response to lipopolysaccharide |
| GOTERM_BP_DIRECT | GO:0070374 | positive regulation of ERK1 and ERK2 cascade |
| GOTERM_BP_DIRECT | GO:0009615 | response to virus |
| GOTERM_BP_DIRECT | GO:0019221 | cytokine-mediated signaling pathway |
| GOTERM_BP_DIRECT | GO:0043410 | positive regulation of MAPK cascade |
| GOTERM_BP_DIRECT | GO:0032760 | positive regulation of tumor necrosis factor production |
| GOTERM_BP_DIRECT | GO:0042127 | regulation of cell population proliferation |
| GOTERM_BP_DIRECT | GO:0035458 | cellular response to interferon-beta |
| GOTERM_BP_DIRECT | GO:0071346 | cellular response to type II interferon |
| GOTERM_BP_DIRECT | GO:0050830 | defense response to Gram-positive bacterium |
| GOTERM_BP_DIRECT | GO:0043123 | positive regulation of canonical NF-kappaB signal transduction |
| GOTERM_BP_DIRECT | GO:0006935 | chemotaxis |
| GOTERM_BP_DIRECT | GO:0045071 | negative regulation of viral genome replication |
| GOTERM_BP_DIRECT | GO:0032729 | positive regulation of type II interferon production |
| GOTERM_BP_DIRECT | GO:0006952 | defense response |
| GOTERM_BP_DIRECT | GO:0001819 | positive regulation of cytokine production |
| GOTERM_BP_DIRECT | GO:0050729 | positive regulation of inflammatory response |
| GOTERM_BP_DIRECT | GO:0032755 | positive regulation of interleukin-6 production |
| GOTERM_BP_DIRECT | GO:0051092 | positive regulation of NF-kappaB transcription factor activity |
| GOTERM_BP_DIRECT | GO:0045785 | positive regulation of cell adhesion |
| GOTERM_BP_DIRECT | GO:0034097 | response to cytokine |
| GOTERM_BP_DIRECT | GO:0050727 | regulation of inflammatory response |
| GOTERM_BP_DIRECT | GO:0002218 | activation of innate immune response |
| GOTERM_BP_DIRECT | GO:0071347 | cellular response to interleukin-1 |
| GOTERM_BP_DIRECT | GO:0032731 | positive regulation of interleukin-1 beta production |
| GOTERM_BP_DIRECT | GO:0034341 | response to type II interferon |
| GOTERM_BP_DIRECT | GO:0060337 | type I interferon-mediated signaling pathway |
| GOTERM_BP_DIRECT | GO:0032757 | positive regulation of interleukin-8 production |
| GOTERM_MF_DIRECT | GO:0003725 | double-stranded RNA binding |
| GOTERM_BP_DIRECT | GO:0032722 | positive regulation of chemokine production |
| GOTERM_BP_DIRECT | GO:0035456 | response to interferon-beta |
| GOTERM_BP_DIRECT | GO:0034612 | response to tumor necrosis factor |
| GOTERM_BP_DIRECT | GO:0010575 | positive regulation of vascular endothelial growth factor production |
| GOTERM_BP_DIRECT | GO:0035455 | response to interferon-alpha |

| **Category** | **Term** | **name** |
| --- | --- | --- |
| KEGG_PATHWAY | hsa04621 | NOD-like receptor signaling pathway |
| KEGG_PATHWAY | hsa04060 | Cytokine-cytokine receptor interaction |
| KEGG_PATHWAY | hsa04061 | Viral protein interaction with cytokine and cytokine receptor |
| KEGG_PATHWAY | hsa05164 | Influenza A |
| KEGG_PATHWAY | hsa04668 | TNF signaling pathway |
| KEGG_PATHWAY | hsa04625 | C-type lectin receptor signaling pathway |
| KEGG_PATHWAY | hsa05169 | Epstein-Barr virus infection |
| KEGG_PATHWAY | hsa05162 | Measles |
| KEGG_PATHWAY | hsa04380 | Osteoclast differentiation |
| KEGG_PATHWAY | hsa05146 | Amoebiasis |
| KEGG_PATHWAY | hsa05160 | Hepatitis C |
| KEGG_PATHWAY | hsa05321 | Inflammatory bowel disease |
| KEGG_PATHWAY | hsa05200 | Pathways in cancer |
| KEGG_PATHWAY | hsa05167 | Kaposi sarcoma-associated herpesvirus infection |
| KEGG_PATHWAY | hsa05163 | Human cytomegalovirus infection |
| KEGG_PATHWAY | hsa05152 | Tuberculosis |
| KEGG_PATHWAY | hsa05133 | Pertussis |
| KEGG_PATHWAY | hsa05202 | Transcriptional misregulation in cancer |
| KEGG_PATHWAY | hsa05165 | Human papillomavirus infection |
| KEGG_PATHWAY | hsa05330 | Allograft rejection |
| KEGG_PATHWAY | hsa04940 | Type I diabetes mellitus |
| KEGG_PATHWAY | hsa04936 | Alcoholic liver disease |
| KEGG_PATHWAY | hsa05332 | Graft-versus-host disease |
| KEGG_PATHWAY | hsa04148 | Efferocytosis |
| KEGG_PATHWAY | hsa05166 | Human T-cell leukemia virus 1 infection |

**Table S3. The complete list of significantly overlapping GO terms and KEGG pathways among three datasets.**

**
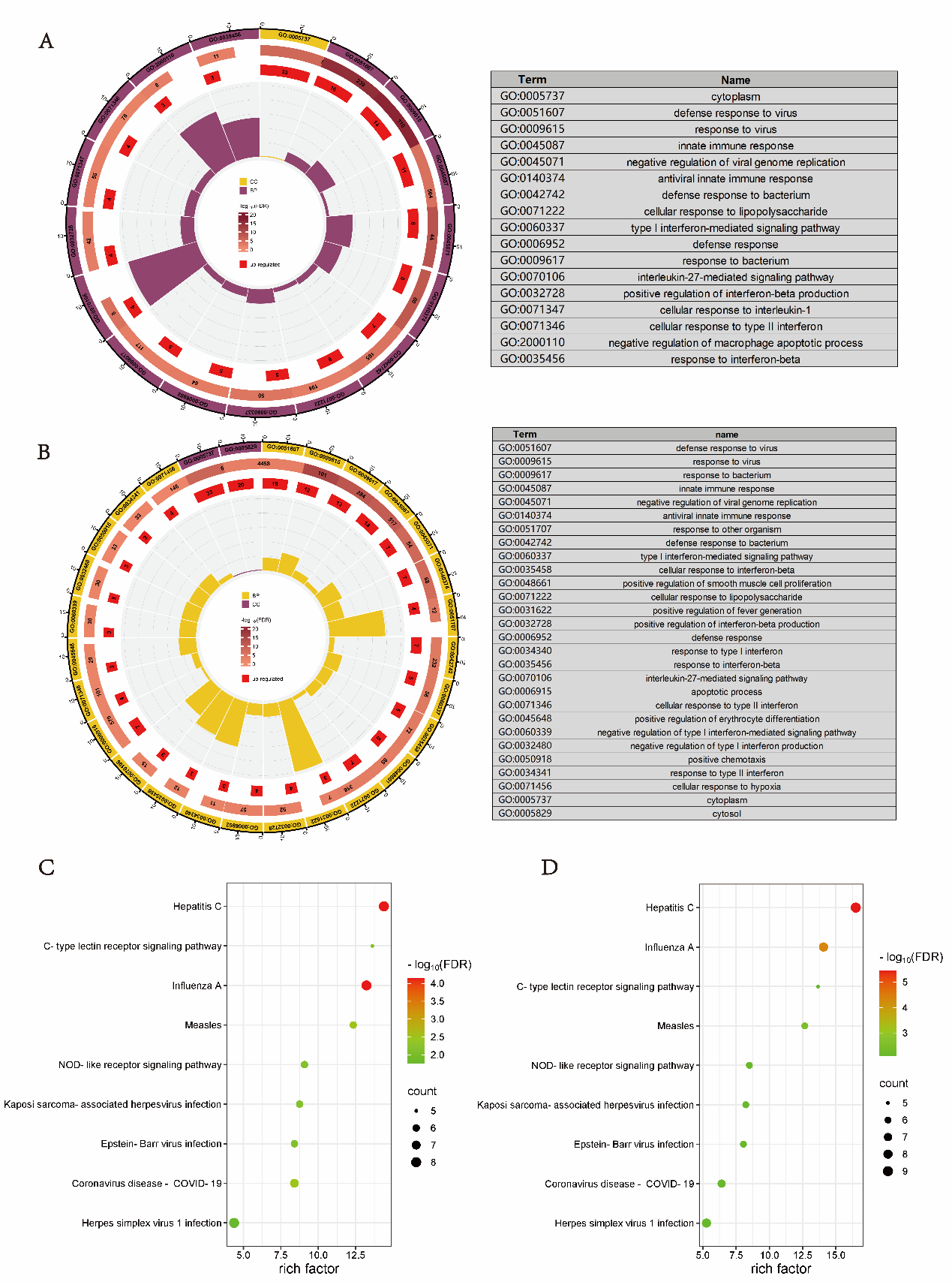
**

**Figure S1.** **Using species-specific annotation resources to perform GO and KEGG analysis on common DEGs.** (**A, B**) GO enrichment analysis with human GO database (**A**) and mouse GO database (**B**). (**C, D**) KEGG enrichment analysis with human KEGG pathway repository (**C**) and mouse KEGG pathway repository (**D**).
