# Supplementary material for: Integrative transcriptomic analysis reveals cross-species conserved core genes and pathways in alveolar macrophages during ALI/ARDS
Source: BMC Pulm Med. 2025 Oct 2;25:447. doi: 10.1186/s12890-025-03928-y (PMC12492643; doi:10.1186/s12890-025-03928-y)
Supplement: Supplementary file 2 — Supplementary material 2. [file 12890_2025_3928_MOESM2_ESM.docx]

**ACOD1/IRG1** **(～55 kDa)**


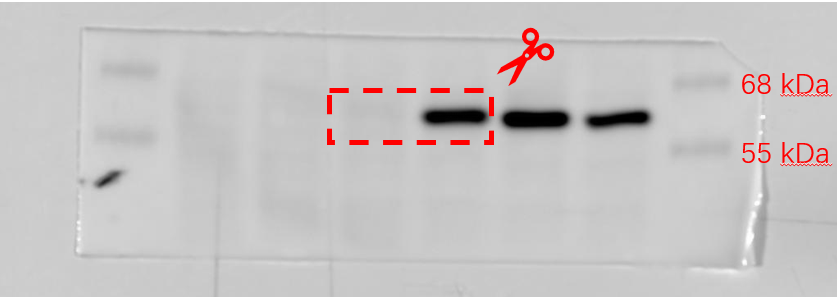


**Vinculin (～124 kDa)**


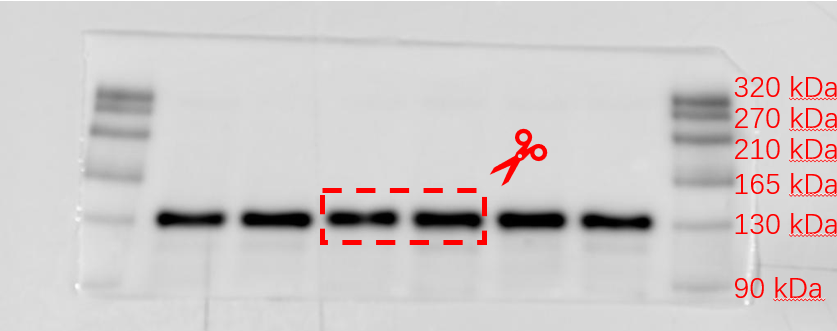


**Prestained Protein Marker**


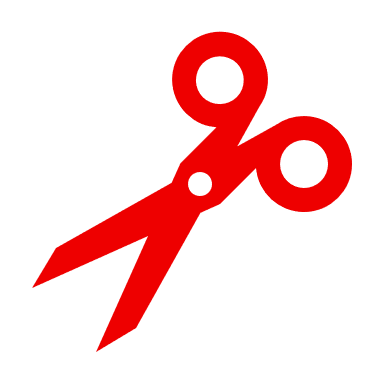

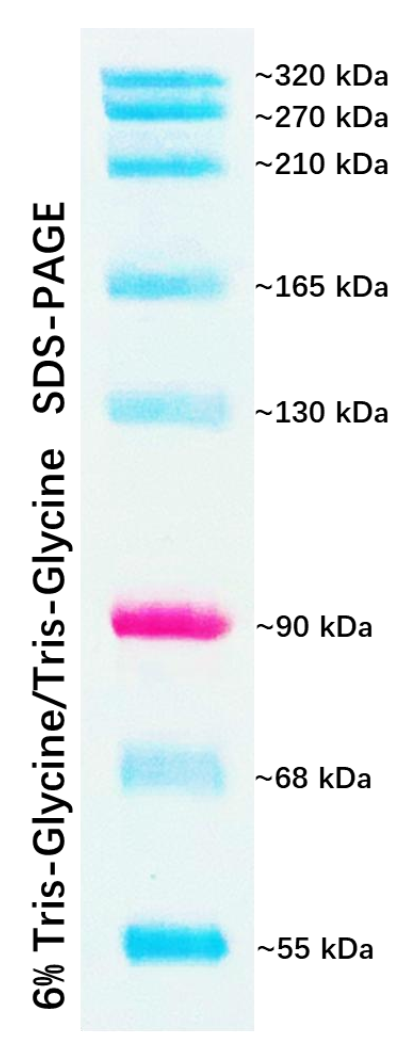


Two groups with three biological replicates each (each lane is a biological replicate), all on the same PVDF membrane. The bands selected by the dotted box are those we cropped for presentation in the paper. Meanwhile, to save the amount of antibodies used, we cropped the whole membrane before incubating it with the primary antibody. The parts indicated by the dotted lines in the example image of the color marker are the positions where we made the cropping.

**TNIP3 (～40 kDa)**


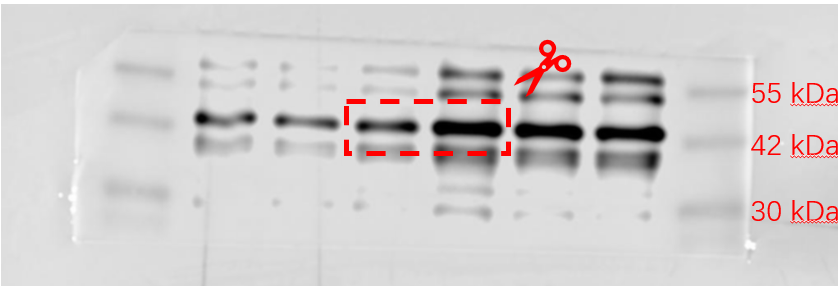


**Vinculin** **(～124 kDa)**


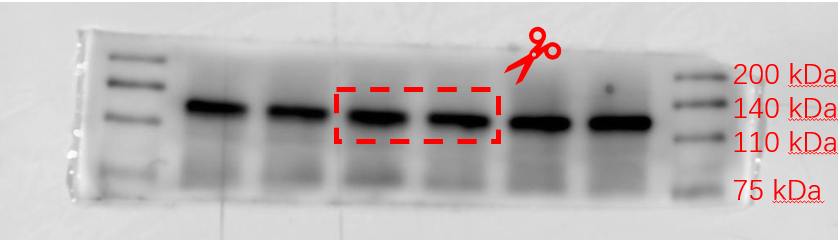


**Prestained Protein Marker**


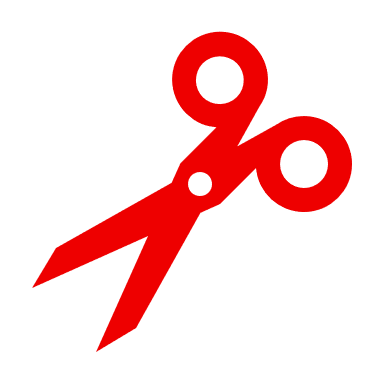

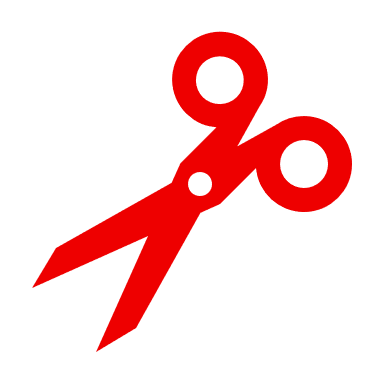

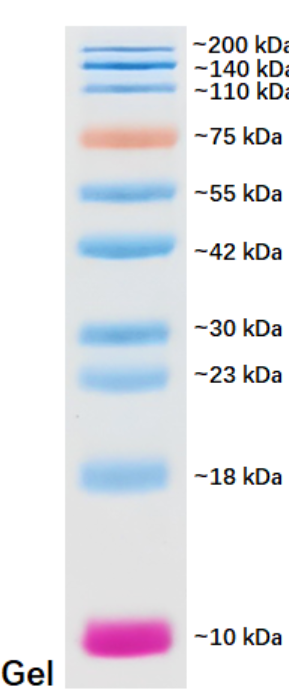


Two groups with three biological replicates each (each lane is a biological replicate), all on the same PVDF membrane. The bands selected by the dotted box are those we cropped for presentation in the paper. Meanwhile, to save the amount of antibodies used, we cropped the whole membrane before incubating it with the primary antibody. The parts indicated by the dotted lines in the example image of the color marker are the positions where we made the cropping.
